# Efficacy, Safety, and Tolerability of Three Regimens for Prevention of Malaria: A Randomized, Placebo-Controlled Trial in Ugandan Schoolchildren

**DOI:** 10.1371/journal.pone.0013438

**Published:** 2010-10-19

**Authors:** Joaniter Nankabirwa, Bonnie Cundill, Sian Clarke, Narcis Kabatereine, Philip J. Rosenthal, Grant Dorsey, Simon Brooker, Sarah G. Staedke

**Affiliations:** 1 Uganda Malaria Surveillance Project, Kampala, Uganda; 2 London School of Hygiene & Tropical Medicine, London, United Kingdom; 3 Vector Control Division, Uganda Ministry of Health, Kampala, Uganda; 4 Department of Medicine, San Francisco General Hospital, University of California San Francisco, San Francisco, California, United States of America; 5 Malaria Public Health and Epidemiology Group, Kenya Medical Research Institute (KEMRI)-Wellcome Trust Research Programme, Nairobi, Kenya; Menzies School of Health Research, Australia

## Abstract

**Background:**

Intermittent preventive treatment (IPT) is a promising malaria control strategy; however, the optimal regimen remains unclear. We conducted a randomized, single-blinded, placebo-controlled trial to evaluate the efficacy, safety, and tolerability of a single course of sulfadoxine-pyrimethamine (SP), amodiaquine + SP (AQ+SP) or dihydroartemisinin-piperaquine (DP) among schoolchildren to inform IPT.

**Methods:**

Asymptomatic girls aged 8 to 12 years and boys aged 8 to 14 years enrolled in two primary schools in Tororo, Uganda were randomized to receive one of the study regimens or placebo, regardless of presence of parasitemia at enrollment, and followed for 42 days. The primary outcome was risk of parasitemia at 42 days. Survival analysis was used to assess differences between regimens.

**Results:**

Of 780 enrolled participants, 769 (98.6%) completed follow-up and were assigned a treatment outcome. The risk of parasitemia at 42 days varied significantly between DP (11.7% [95% confidence interval (CI): 7.9, 17.1]), AQ+SP (44.3% [37.6, 51.5]), and SP (79.7% [95% CI: 73.6, 85.2], p<0.001). The risk of parasitemia in SP-treated children was no different than in those receiving placebo (84.6% [95% CI: 79.1, 89.3], p = 0.22). No serious adverse events occurred, but AQ+SP was associated with increased risk of vomiting compared to placebo (13.0% [95% CI: 9.1, 18.5] vs. 4.7% [95% CI: 2.5, 8.8], respectively, p = 0.003).

**Conclusions:**

DP was the most efficacious and well-tolerated regimen tested, although AQ+SP appears to be a suitable alternative for IPT in schoolchildren. Use of SP for IPT may not be appropriate in areas with high-level SP resistance in Africa.

**Trial Registration:**

ClinicalTrials.gov NCT00852371

## Introduction

Despite renewed global commitment to malaria control and substantial increases in funding, the burden of malaria in Africa remains high [1]. Typically, malaria control efforts focus on young children and pregnant women, who bear the greatest burden of morbidity and mortality. Older children are generally protected from the most serious effects of illness by antimalarial immunity acquired through repeated infections [2]. However, malaria in school-aged children remains common and can substantially reduce school attendance, cognition, and learning [3]. Older children also serve as a major reservoir of parasites, contributing to transmission in the wider community. Yet surprising little is known about how best to control malaria in schools [4], [5].

Available and proven malaria control interventions include insecticide-treated bed nets (ITNs), indoor residual spraying (IRS) of insecticide, and effective case management. However, even with ITNs and effective management with artemisinin-based combination therapies (ACTs), children living in highly endemic areas are frequently infected [6]. Intermittent preventive treatment (IPT), the administration of curative doses of antimalarial treatment at predefined intervals regardless of infection status, is gaining momentum as a malaria control strategy [7]. IPT has been shown to benefit infants [8], children in areas of seasonal malaria transmission [9]-[12], and older school-aged children [13], [14], and the World Health Organization recently released a new policy on IPT in infants [15]. However, a number of scientific and operational issues for IPT remain to be determined, and the optimal regimen remains unclear [16]. Sulfadoxine-pyrimethamine (SP) has been most widely studied for IPT and is currently recommended in pregnancy, but there is evidence that IPT with SP in pregnancy is ineffective in areas of high SP resistance [17], [18]. The efficacy and safety of alternatives for IPT is little studied.

We conducted a randomized, single-blinded, placebo-controlled trial, modeled on standard antimalarial drug efficacy trials, to evaluate three regimens available for IPT in Ugandan schoolchildren: SP, amodiaquine combined with SP (AQ+SP), and dihydroartemisinin-piperaquine (DP). The primary outcome was the 42-day risk of parasitemia. Our aim was to compare the efficacy of the regimens for eliminating asymptomatic infections and preventing new infections, and to assess the safety, tolerability, and acceptability of the regimens, after a single course of treatment. The inclusion of the placebo arm allowed us to evaluate the efficacy of SP among asymptomatic children with acquired immunity in an area with substantial resistance, and to assess the risk of adverse events with each of the regimens.

## Methods

The protocol for this trial and supporting CONSORT checklist are available as supporting information; see Checklist S1 and Protocol S1.

### Ethics

The study was approved by the Ugandan National Council for Science and Technology, the ethics committees of Makerere University and the London School of Hygiene and Tropical Medicine, and the Ugandan National Drug Authority. The trial was overseen by an independent Data and Safety Monitoring Board. Permissions were obtained from district officials and teachers.

### Study site

The trial was conducted between February and July 2008 in two primary schools in Tororo district, Uganda, where the estimated entomological inoculation rate in 2001-02 was 562 [19], and up to 60% of children were parasitemic in 2008 [20]. In prior studies of symptomatic patients, the risk of recrudescence at 28 days was 35% with SP in Kampala [21], and 18% with AQ+SP [22] and 0.3% with DP in Tororo [23]. The prevalence of parasite genetic polymorphisms associated with decreased sensitivity to both SP and AQ is high in Uganda. The five mutations that mediate an intermediate level of resistance to SP (dhfr N51I, C59R, and S108N and dhps A437G and K540E) are very common in Tororo (over 80% prevalence) [24]; however, the dhfr I164L and dhps A581G mutations, which mediate higher-level resistance, are uncommon [25]. For AQ, the pfcrt 76T mutation is fixed at nearly 100% prevalence, and the pfmdr1 N86Y and D1246Y mutations are also over 80% in Tororo [26].

In 2004, the Ugandan Ministry of Health selected the combination of artemether-lumefantrine to replace chloroquine + SP as first-line treatment for uncomplicated malaria. Although the new antimalarial policy was adopted in 2004, it was not fully implemented until 2006, and non-recommended regimens continue to be used frequently in Uganda due to stock-outs of artemether-lumefantrine [27].

### Participants

School meetings were held to explain the study and written informed consent was sought from parents/guardians of girls aged 8 to 12 years, and boys aged 8 to 14 years, enrolled in classes 1 to 7. Assent to participate was sought from all children. Additional selection criteria included: no known allergy or prior adverse reaction to study medications; no onset of menstruation; no fever (axillary temperature <37.5°C) or history of fever in previous 24 hours; no evidence of severe malaria or danger signs; no ongoing antimalarial treatment; hemoglobin >7.0 g/dL; and parasite density ≤10,000/µl.

### Enrollment procedures

At enrollment (day 0), we conducted a standardized assessment of symptoms, which served as the baseline for monitoring of subsequent adverse events. A physical examination was conducted, including measurement of temperature, height and weight. A fingerprick blood sample was obtained for hemoglobin measurement, thick and thin blood smears, and to store on filter paper.

### Interventions

On day 0, children were randomly assigned to receive SP (Fansidar, Roche, 500 mg/25 mg tablets, 25 mg/kg sulfadoxine and 1.25 mg/kg pyrimethamine per treatment as a single dose), AQ+SP (Camoquin, Parke-Davis, 200 mg tablets, 10 mg/kg on days 0 and 1, and 5 mg/kg on day 2), DP (Duocotexin, Holley Pharm, 40 mg dihydroartemisinin/320 mg piperaquine tablets targeting a total dose of 6.4 and 51.2 mg/kg of dihydroartemisinin and piperaquine, respectively, given in 3 equally divided daily doses to the nearest ¼ tablet), or placebo (Cosmos Limited, Nairobi, Kenya) administered to simulate the amodiaquine dosing schedule. Children in the SP group also received placebo tablets on days 1 and 2.

### Randomization and treatment administration

Randomization codes were computer-generated in blocks of eight by an investigator not directly involved in the project and were sealed in numbered envelopes. The study nurse assigned treatment numbers sequentially and allocated treatments by opening the envelope corresponding to the treatment number. All other study personnel were blinded to treatment assignments. Children were not informed of their treatment regimen, but the color and taste of study medications were dissimilar. All treatment was directly observed. After drug administration, children were observed for 30 minutes and treatment was re-administered if vomiting occurred. No child vomited more than once.

### Follow-up visits

Children were followed-up at school on days 1, 2, 3, 7, 14, 28 and 42, and any additional day that they felt ill. Children absent for scheduled follow-up were visited at home. Follow-up evaluations consisted of a standardized history and physical examination. Fingerprick blood samples were taken on days 7, 14, 28, 42, (and on any unscheduled day that fever was reported) to repeat thick blood smears and for storage on filter paper. Hemoglobin was reassessed on day 42. Children with a hemoglobin <10 g/dL were treated with ferrous sulfate for 14 days according to the Integrated Management of Childhood Illnesses guidelines. Acceptability of the study regimens was assessed using a semi-structured questionnaire administered on day 7. Children were excluded if consent to participate was withdrawn or if they were lost to follow-up. Children who received antimalarial drugs outside of the study were followed for 42 days but were not assigned an efficacy outcome.

Rescue therapy with artemether-lumefantrine was provided for danger signs or severe malaria with parasitemia; parasite density >10,000/µl; fever (axillary temperature >37.5°C) or history of fever in the previous 24 hours on days 3 to 42 with parasitemia; presence of parasitemia on day 42. All children who received rescue therapy were followed for the full 42 days. Children found to have illnesses other than malaria received standard of care, or were referred appropriately.

### Laboratory evaluations

Blood smears were stained with 2% Giemsa for 30 minutes. Parasite densities were determined from thick blood smears by counting the number of asexual parasites per 200 white blood cells (or per 500 if the count was less than 10 parasites/200 white cells), assuming a white blood cell count of 8,000/µl. A smear was considered negative after reviewing 100 high-powered fields. Gametocytemia was also determined using similar methodology. Thin blood smears were reviewed for species identification. Two independent microscopists read the slides, with a third microscopist resolving discrepant results. Because microscopy results were generally not available until the following day, a child could be excluded after randomization and treatment if the parasite density was >10,000/µl. Hemoglobin concentration was measured using a portable spectrophotometer (HemoCue, Ängelholm, Sweden).

Molecular genotyping techniques were used to distinguish recrudescent from new infections in children who were parasitemic at baseline and developed recurrent parasitemia between days 3 and 42. DNA was isolated from filter paper blood samples collected at enrollment and on the day of recurrent parasitemia using chelex extraction. Paired samples were genotyped in a stepwise fashion using *msp-2*, *msp-1*, and four microsatellites [28]. If, for any of the six loci, an allele was not shared between day 0 and day of recurrence, the infection was classified as a new infection. If at least one allele was shared between day 0 and day of recurrence at all six loci, the infection was classified as a recrudescence.

### Sample size and outcomes

Sample size was calculated to test the hypothesis that treatment with AQ+SP or DP would decrease the 42-day risk of parasitemia (the primary outcome) compared to treatment with SP, assuming a risk of 65% in SP-treated children. We calculated that 190 children per arm would be needed to detect a significant difference of ≥15% (p<0.05, two-sided test), with 80% power, allowing for 10% loss to follow-up [29]. No formal adjustments for multiple comparisons were made. Secondary outcomes included risks of recrudescence and new infection (adjusted by genotyping), and change in mean hemoglobin from Day 0 to Day 42. Safety outcomes included risks of common and serious adverse events.

### Statistical methods

Data were entered and verified using Epi-Info version 6.04 and analyzed using STATA version 10.0 (STATA Corporation, College Station, TX, USA). The 42-day risk of parasitemia was estimated using Kaplan-Meier survival techniques with corresponding 95% confidence intervals (CI) calculated using Greenwood variance estimates [30]. Children who were excluded or received antimalarials outside the study were censored on the day that these events were identified. Risk differences were estimated and formal hypothesis testing done using unpaired z-tests. A Cox's Proportional Hazards model was fitted to adjust for baseline imbalances in the presence of parasitemia. Since we expected the rate of parasitemia to differ between regimens over the study period due to varying drug half-lives, the model was fitted with an interaction between treatment regimen and time band (0–14 days, 15–28 days, 29–42 days), assuming proportional hazards within each time band [30].

The proportions of children receiving rescue therapy were compared using the chi-squared test. The risks of recrudescence and new infections were also estimated using Kaplan-Meier techniques, as described above. When estimating the risk of recrudescence only children who were parasitemic at baseline were included in the analysis and those children experiencing a new infection were censored on the day of the infection. When estimating the risk of new infection only children who were not parasitemic at baseline were included in the analysis. Two-sided t-tests were used to compare the mean change in hemoglobin between days 0 and 42.

An adverse event was defined as any untoward medical occurrence, irrespective of its suspected relationship to the study medications. All events were graded by severity and relationship to study treatment [31]. The risk of experiencing an adverse event was estimated using Kaplan-Meier techniques, censoring for those who received rescue therapy. Comparisons with placebo were made using z-tests. Quantitative data on acceptability of different treatment regimens were compared to those of placebo using chi-square and Fisher's exact tests.

## Results

### Participant flow

**Figure 1:**
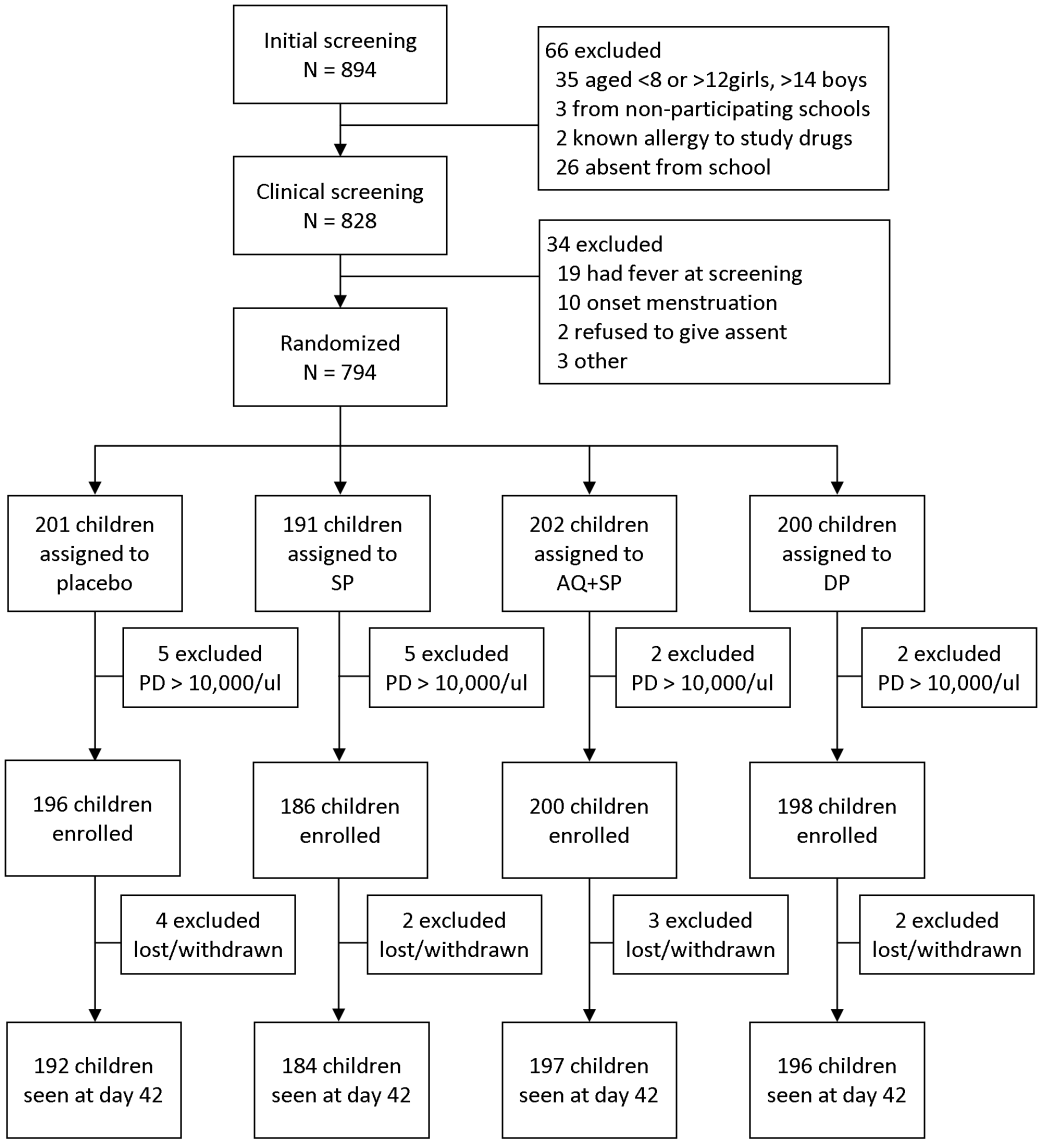
Trial profile. SP  =  sulfadoxine-pyrimethamine; AQ+SP  =  amodiaquine + sulfadoxine-pyrimethamine; DP  =  dihydroartemisinin-piperaquine; PD  =  parasite density.

Of 894 children initially screened, 794 (88.8%) met the inclusion criteria and were randomized to one of the treatment arms (Figure 1). Fourteen children had a parasite density of >10,000/µl and were excluded after randomization. Of the 780 children enrolled, 769 (98.6%) completed follow-up and were included in the primary outcome analysis. The characteristics of participants randomized to each treatment were similar, although children receiving AQ+SP were less likely to be parasitemic (Table 1). Just over half (399 [51.3%]) of children were parasitemic at enrollment. Nearly all infections were *P. falciparum*.

**Table 1:** Baseline characteristics by treatment regimen.

Characteristic	Treatment regimen (N = 780)			
	Placebo	SP	AQ+SP	DP
Number of children	196	186	200	198
Gender (n female, %)	78 (39.8)	70 (37.6)	78 (39.0)	87 (43.9)
Mean age (SD, years)	10.6 (1.86)	10.6 (1.85)	10.7 (1.93)	10.3 (1.73)
Mean hemoglobin (SD, g/dL)	12.7 (1.38)	12.7 (1.31)	12.7 (1.33)	12.4 (1.26)
Bednet use* † (%)	57 (29.1)	47 (25.3)	56 (28.0)	56 (28.2)
Parasitemia ‡ (%)	110 (56.4)	98 (52.7)	87 (43.5)	104 (52.8)
*―P. falciparum*	110	95	87	103
*―P. falciparum/P. malariae*	0	1	0	0
*―P. malariae*	0	1	0	1
―Not known	0	1	0	0
Gametocyte presence ‡ (%)	22 (11.3)	14 (7.5)	17 (8.5)	11 (5.6)
Geometric mean parasite density per µl ‡ (95% CI)	338.8 (255.9, 448.4)	409.6 (291.8, 574.3)	477.1 (333.1, 683.4)	325.5 (234.4, 451.0)

*Children who slept under a bednet last night.

† Two children were missing bednet data; 1 in SP and 1 in DP.

‡ Two children were missing blood smear data; 1 in placebo and 1 in DP.

### 42-day risk of parasitemia

At day 42, there was no evidence that SP provided any benefit over placebo (Table 2) even after adjusting for the presence of baseline parasitemia (data not shown). The risk of parasitemia in children treated with DP and AQ+SP was significantly lower than in those receiving SP, and DP was superior to AQ+SP (risk difference 32.6 [95% CI 24.3, 40.9]; p<0.001).

**Table 2:** Risk of parasitemia at 42 days associated with the treatment regimens as compared to SP.

Treatment	n/N	% Risk	Risk difference	p-value
		(95% CI)	(95% CI)	
SP	147/186	79.7 (73.6, 85.2)	—	—
Placebo*	164/196	84.6 (79.1, 89.3)	−4.9 (−12.6, 2.9)	0.22
AQ+SP	87/200	44.3 (37.6, 51.5)	35.4 (26.3, 44.5)	<0.001
DP	23/198	11.7 (7.9, 17.1)	68.0 (60.6, 75.4)	<0.001

*One child in the placebo arm was lost to follow up on day 0 and hence was censored on this day.

### Risk of rescue therapy

Of 421 children who were parasitemic during follow-up, only 30 (7.1%) developed symptomatic malaria, requiring rescue therapy with artemether-lumefantrine (13 in the placebo group, 13 SP, 3 AQ+SP, and 1 DP). Only one child, who received placebo, developed symptoms within 7 days of treatment (on day 3). An additional 24 (5.7%) children received rescue therapy for a parasite density >10,000/µl in the absence of fever (14 placebo, 7 SP, 2 AQ+SP, and 1 DP). The overall risk of rescue therapy was significantly lower in children receiving AQ+SP (2.5%, p = 0.001) or DP (1.0%, p<0.001), than in those treated with SP (10.7%).

### Risk of recrudescence

**Figure 2:**
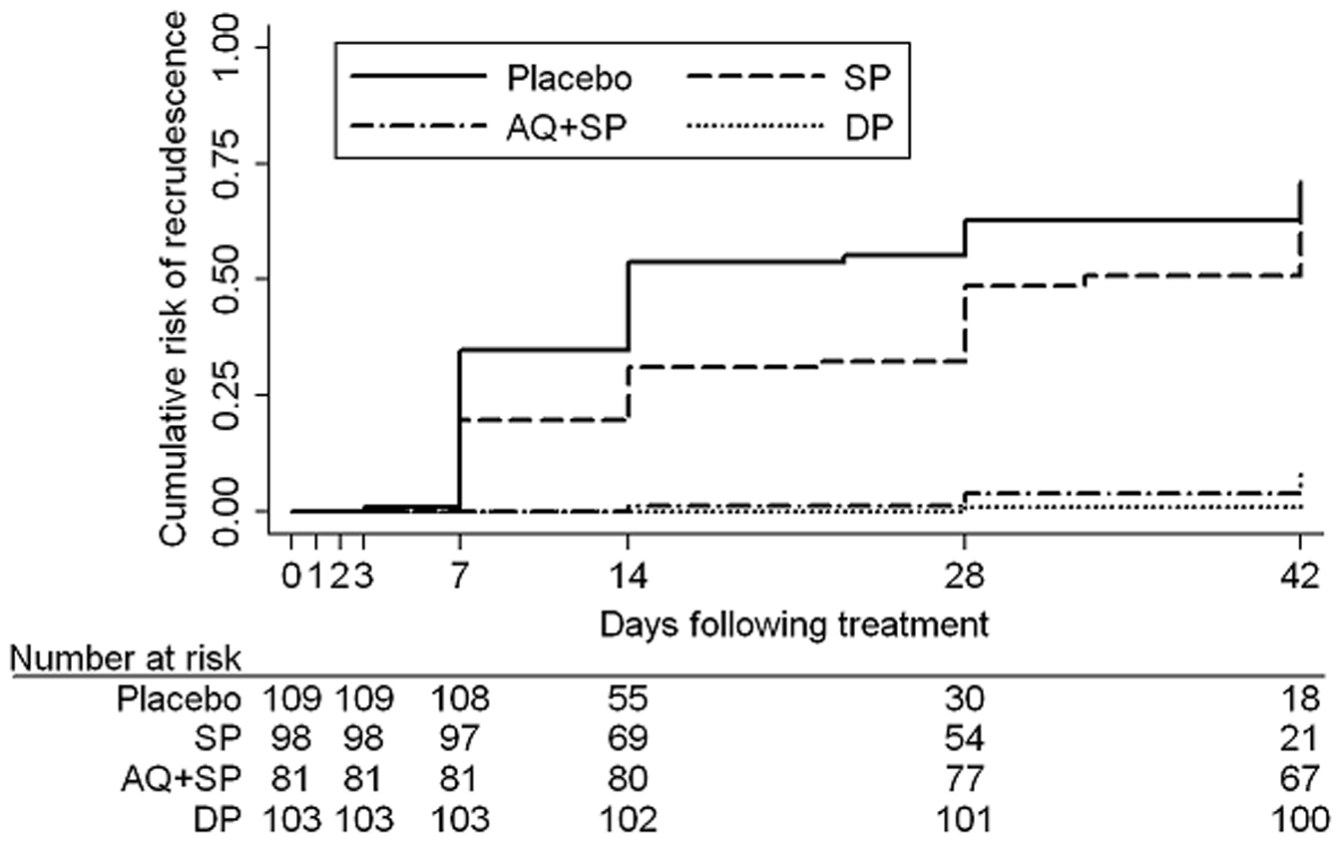
Cumulative risk of recrudescence in children with malaria parasitemia at baseline over 42 days by treatment regimen. SP  =  sulfadoxine-pyrimethamine; AQ+SP  =  amodiaquine + sulfadoxine-pyrimethamine; DP  =  dihydroartemisinin-piperaquine.

Of 399 children who were parasitemic at baseline, 228 were parasitemic during follow-up. The child who received rescue therapy on day 3 was classified as a recrudescence. Genotyping results were available for 221 children; genotyping was unsuccessful in 6 cases, and results were missing for one. Thus, 392 children were included in the analysis for risk of recrudescence. There were 122 recrudescences, 99 new infections, and 171 who remained free of parasites. Children treated with AQ+SP and DP were at significantly lower risk of recrudescence than those receiving SP (Figure 2, Table 3). There was no evidence that treatment with SP provided any benefit over placebo.

**Table 3:** Risk of recrudescence in children with parasites on Day 0 (adjusted by genotyping) and risk of new infection in children who were free of parasites on Day 0.

Treatment	n/N	% Risk (95% CI)	Risk difference (95% CI)	p-value
**Risk of recrudescence in children with parasites on day 0 (n = 392)**				
SP	50/98	65.0 (52.8, 76.9)	—	—
Placebo	64/109	71.0 (60.1, 81.2)	−6.1 (−22.4, 10.3)	0.47
AQ+SP	6/82	8.1 (3.7, 17.2)	56.8 (43.0, 70.7)	<0.001
DP	2/103	2.0 (0.5, 7.7)	63.0 (50.4, 75.6)	<0.001
**Risk of new infection in children who were free of parasites on day 0**				
SP	62/88	70.4 (60.8, 79.6)	—	—
Placebo	64/85	75.3 (65.8, 83.9)	−4.8 (−18.1, 8.4)	0.47
AQ+SP	55/113	49.1 (40.3, 58.7)	21.4 (8.1, 34.7)	0.002
DP	12/93	13.1 (7.7, 21.9)	57.4 (45.6, 69.1)	<0.001

### Risk of new infection

**Figure 3:**
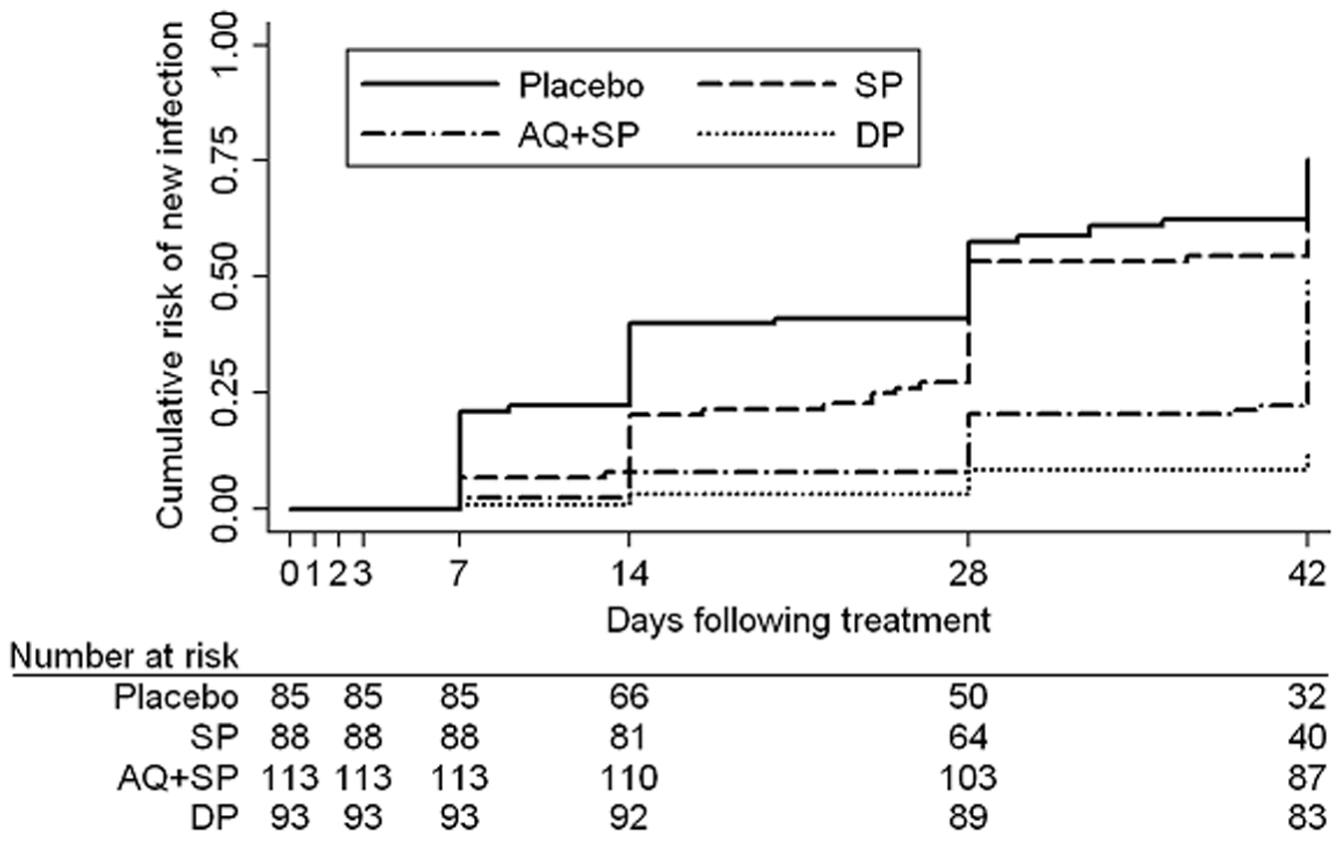
Cumulative risk of new infection in children free of malaria parasites at baseline over 42 days by treatment regimen. SP  =  sulfadoxine-pyrimethamine; AQ+SP  =  amodiaquine + sulfadoxine-pyrimethamine; DP  =  dihydroartemisinin-piperaquine.

Of 379 children who were free of parasites at baseline, 193 became parasitemic during follow-up and were classified as new infections. Again, there was no evidence that SP provided any benefit over placebo for prevention of new infections (Figure 3, Table 3). Both AQ+SP and DP were more efficacious than SP, but DP was superior for preventing new infections (DP vs. AQ+SP: risk difference 36.0 [95% CI: 24.5, 47.6] p<0.001).

### Impact on hemoglobin

Mean hemoglobin fell between day 0 and day 42 in children treated with SP (−0.18 g/dL [95% CI: −0.38, 0.02]); the mean change in SP-treated children was no different than in those receiving placebo (−0.24 g/dL [95% CI: −0.44, −0.05], p = 0.65). In contrast, day 42 hemoglobin in children receiving AQ+SP and DP was substantially higher than at baseline (increasing by 0.37 g/dL [95% CI: 0.18, 0.56] and 0.34 g/dL [95% CI: 0.15, 0.53], respectively), and the mean change was significantly greater than in SP-treated children (p<0.001 for both comparisons). There was no evidence that mean change in hemoglobin was different in children treated with AQ+SP and DP (difference 0.03 [95% CI −0.24, 0.30] p = 0.82).

### Adverse events

By day 42, 973 adverse events were reported by 477 (61%) children, including 897 (92%) mild events, 76 (8%) moderate and no serious adverse events (Table 4). Overall, the risk of experiencing any adverse event did not differ between the regimens at day 42. However, within the first 3 days of treatment, this risk was higher in children receiving AQ+SP than in those receiving placebo (44.4% [95% CI: 37.8, 51.6] vs. 30.3% [95% CI: 24.4, 37.2], respectively, p = 0.003). The most common adverse events reported were headache, cough, abdominal pain, coryza, skin rash, nausea, vomiting, and diarrhea. Children treated with AQ+SP were at significantly higher risk of vomiting than those receiving placebo, which was greatest within the first 3 days after treatment. There was also a non-significant trend toward increased risk of nausea associated with AQ+SP, which was greatest soon after treatment (data not shown). Children treated with AQ+SP or DP were significantly less likely to report a history of fever or to be febrile than those receiving placebo.

**Table 4:** Risk of adverse events at 42 days in all participants by treatment group, and pairwise comparisons with placebo.

	Placebo (N = 196)	SP (N = 186)		AQ+SP (N = 200)		DP (N = 198)	
	% Risk (95% CI)	% Risk (95% CI)	p-value	% Risk (95% CI)	p-value	% Risk (95% CI)	p-value
**Any adverse event**	63.5 (56.6, 70.3)	61.2 (54.2, 68.4)	0.66	60.8 (54.1, 67.6)	0.59	59.1 (52.3, 66.0)	0.38
Fever/History of fever	7.9 (4.9, 12.8)	5.0 (2.7, 9.5)	0.26	3.5 (1.7, 7.2)	0.06	3.0 (1.4, 6.6)	0.03
Headache	24.3 (18.9, 31.0)	26.1 (20.4, 33.2)	0.68	25.5 (20.0, 32.1)	0.79	21.2 (16.2, 27.6)	0.47
Abdominal Pain	19.0 (14.2, 25.3)	16.9 (12.2, 23.2)	0.59	18.0 (13.3, 24.1)	0.80	17.7 (13.0, 23.8)	0.73
Nausea	7.2 (4.3, 11.9)	6.5 (3.8, 11.2)	0.80	12.5 (8.6, 17.9)	0.08	5.1 (2.8, 9.2)	0.37
Vomiting	4.7 (2.5, 8.8)	2.8 (1.2, 6.6)	0.34	13.0 (9.1, 18.5)	0.003	5.1 (2.8, 9.2)	0.87
Diarrhea	3.3 (1.5, 7.2)	3.3 (1.5, 7.2)	0.97	4.5 (2.4, 8.5)	0.53	5.1 (2.7, 9.2)	0.38
Cough	27.1 (21.3, 34.0)	17.2 (12.4, 23.5)	0.021	17.0 (12.5, 23.0)	0.017	17.2 (12.6, 23.2)	0.019
Coryza	13.3 (9.2, 19.0)	12.4 (8.3, 18.2)	0.80	9.0 (5.8, 13.9)	0.18	14.2 (10.0, 19.8)	0.80
Skin Rash/Pruritis	6.4 (3.7, 11.0)	8.2 (5.0, 13.2)	0.51	2.5 (1.1, 5.9)	0.06	5.6 (3.1, 9.8)	0.73

Children were more likely to rate the taste of the tablets as ‘poor’ or ‘fair’ (than ‘good’ or ‘excellent’) if they were treated with AQ+SP (67/199 [33.7%]) or DP (55/198 [27.8%]) than those receiving placebo (20/195 [10.3%], p<0.001). However, most children reported that they would be willing (657/775 [84.8%]) or very willing (62/775 [8.0%]) to take the tablets each school term, regardless of treatment group (94% placebo, 96% SP, 90% AQ+SP, 91% DP).

## Discussion

Intermittent preventive treatment of schoolchildren is a promising malaria control strategy; however, with increasing SP resistance the optimal drug regimen remains unclear. To offer insights into the relative benefits of IPT with different antimalarial regimens, we evaluated the impact of a single treatment in Ugandan schoolchildren. We found a rank order in the efficacy of study regimens, DP > AQ+SP > SP, with SP providing no benefit over placebo. DP and AQ+SP were also associated with a marked improvement in hemoglobin levels. All regimens were safe and acceptable, although children treated with AQ+SP were at higher risk of vomiting, particularly early after treatment, suggesting that this regimen was less well-tolerated. Our findings suggest that DP will be highly efficacious for IPT in schoolchildren, and that AQ+SP may be a suitable alternative, but that SP should not be used for IPT in areas with high-level SP resistance, as occurs in our study site.

Of the regimens available for IPT, SP has several advantages that make it attractive including low cost, documented safety, simple dosing and a relatively long elimination half-life. However, resistance to SP has become widespread in Africa, which could limit the utility of this regimen [32]. Benefits of IPT with SP have been demonstrated in pregnant women [33], infants [8], and children [11]. However, recent studies conducted in areas with high-level SP resistance suggest that the drug offered no benefit when administered for IPT in pregnant women [17], [18], and infants [34]. The threshold level of resistance beyond which IPT with SP ceases to be effective is unknown, but even when resistance is prevalent, SP may still have a role [33]. Host immunity, which in endemic areas increases with age, is likely to modify the relationship between resistant parasites and treatment outcome [24]. SP may remain effective for prevention of malaria in older children and adults with relatively high levels of acquired antimalarial immunity. However, in our study area, where resistance is common, SP provided no benefit to older children.

DP, a newer ACT regimen, was the most efficacious and best-tolerated regimen in this study. In Senegal, piperaquine combined with dihydroartemisinin (DP) and with SP (SP+P) administered to children under five for IPT during the high transmission season were as effective as AQ+SP for preventing malaria, and were better tolerated [12]. In Mali, two other ACTs, artesunate + SP (AS+SP) or amodiaquine + artesunate (AQ+AS), administered to schoolchildren during the malaria transmission season reduced clinical malaria incidence, asymptomatic parasitemia, and anemia [14]. However, use of an ACT regimen for IPT may not be desirable if an ACT is used as first-line treatment of uncomplicated malaria, as is currently the case in most of sub-Saharan Africa, including Uganda. Tolerance to artemisinins has already been reported in South East Asia [35], and limiting the emergence and spread of artemisinin resistance is of utmost importance [36]. Using a non-ACT regimen, such as AQ+SP, may be preferable for IPT, allowing ACT regimens to be reserved for treatment of symptomatic malaria. We found that AQ+SP was more efficacious than expected given the poor performance of SP, which is consistent with the results of other studies evaluating AQ+SP for treatment [37], [38] and prevention [10], [13]. Provision of AQ+SP for IPT once a school term (three times in one year) to Kenyan schoolchildren reduced the rates of anemia by half and improved children's ability to concentrate in class [13]. Although use of AQ+SP is likely to be increasingly limited by resistance [39], it might be an option in areas where resistance to both drugs is relatively low, particularly in West Africa, where AQ+SP remains highly effective [10], [40].

Safety and tolerability of regimens to be used in IPT programs is a key issue, as they will be administered to asymptomatic children. Serious toxicity has been associated with SP and AQ, including severe cutaneous reactions with SP, and neutropenia and hepatotoxicity with AQ, mainly with long-term chemoprophylaxis [41]–[43]. However, both drugs appear to be safer when used in short-term treatment regimens [44], [45]. In our study, no serious adverse events occurred and all regimens appeared to be safe. Children treated with AQ+SP were at increased risk of early adverse events, particularly vomiting, suggesting that this regimen was less well-tolerated. However, most children assigned to AQ+SP reported willingness to take the medications, suggesting that adverse events may not affect adherence to this regimen. An association between AQ+SP and vomiting was also observed in Senegal [12]. Notably, we did not find an association between AQ+SP and risk of fatigue or weakness, which has previously been reported in studies from Rwanda and Uganda [46], [47].

This trial had several important limitations. Firstly, we only evaluated the effect of a single episode of treatment, limiting inferences regarding the efficacy and safety of repeated doses. Secondly, efficacy of the treatments was measured in terms of risk of parasitemia, rather than incidence of clinical malaria, anemia, or school performance, more typical endpoints for IPT studies. Although we aim to inform IPT, this study is not a direct assessment of IPT. Finally, our study was conducted in an area of very high malaria transmission, and our results may not be generalizable to regions with lower transmission intensity, and thus lower antimalarial immunity.

In summary, DP and AQ+SP were both highly effective in eliminating asymptomatic infections when administered as a single dose to Ugandan schoolchildren, whereas SP alone was not efficacious. DP was superior for preventing new infections. Our results suggest that DP would be appropriate for use in IPT programmes, but AQ+SP may be a suitable alternative in areas where resistance to the individual drugs remains low. Future research is needed to address additional operational questions regarding IPT in schoolchildren, including optimal dosing schedules in different settings, methods of delivery, and integration of IPT with other existing school programs.

## Supporting Information

Protocol S1 Study protocol.
(0.86 MB PDF)

Checklist S1 CONSORT checklist for a randomised trial.
(0.05 MB PDF)
