# Supplementary material for: Efficacy, Safety, and Tolerability of Three Regimens for Prevention of Malaria: A Randomized, Placebo-Controlled Trial in Ugandan Schoolchildren
Source: PLoS One. 2010 Oct 19;5(10):e13438. doi: 10.1371/journal.pone.0013438 (PMC2957410; doi:10.1371/journal.pone.0013438)
Supplement: Protocol S1 — Study protocol. (0.86 MB PDF) [file pone.0013438.s001.pdf]

THE REPUBLIC OF UGANDA

UCSF

IPT in schoolchildren: Comparison of the efficacy, safety, and tolerability of antimalarial regimens

Sponsored by:
Gates Malaria Partnership

Uganda Malaria Surveillance Project
London School of Hygiene & Tropical Medicine
Vector Control Division, Uganda Ministry of Health

Protocol version 1.3
17 March 2008

GatesMalariaPartnership

LONDON SCHOOL OF HYGIENE & TROPICAL MEDICINE

TABLE OF CONTENTS

STUDY SUMMARY
ABBREVIATIONS AND ACRONYMS

1.0 BACKGROUND
- **1.1** INTRODUCTION
- **1.2** BURDEN OF MALARIA IN UGANDA
- **1.3** INTERMITTENT PREVENTIVE TREATMENT
- **1.4** IPT IN SCHOOLCHILDREN
- **1.5** ROLE OF THE EDUCATION SECTOR
- **1.6** CHOICE OF DRUGS FOR IPT
- **1.7** IPT REGIMENS
- **1.8** SAFETY AND TOLERABILITY OF IPT REGIMENS

2.0 RATIONALE

3.0 STUDY OBJECTIVES
- **3.1** PRIMARY OBJECTIVE
- **3.2** SECONDARY OBJECTIVES

4.0 STUDY DESIGN / METHODS
- **4.1** OVERALL STUDY DESIGN
- **4.2** CLASSIFICATION OF TREATMENT OUTCOME
- **4.3** OUTCOME MEASURES

5.0 PARTICIPANT SELECTION AND ENROLLMENT
- **5.1** STUDY SITE
- **5.2** SELECTION CRITERIA
- **5.3** INITIAL RECRUITMENT
- **5.4** SCREENING AND ENROLLMENT

6.0 STUDY INTERVENTION
- **6.1** RANDOMIZATION
- **6.2** TREATMENT ASSIGNMENT AND ALLOCATION
- **6.3** STUDY TREATMENTS
- **6.4** DOSING OF STUDY DRUGS
- **6.5** BLINDING
- **6.6** ADMINISTRATION OF STUDY DRUGS

7.0 FOLLOW-UP EVALUATIONS AND PROCEDURES
- **7.1** SCHEDULED FOLLOW-UP PROCEDURES
- **7.2** UNSCHEDULED FOLLOW-UP
- **7.3** MANAGEMENT OF MALARIA
- **7.4** MANAGEMENT OF NON-MALARIA ILLNESSES

7.5 EVALUATION OF ACCEPTIBILITY
7.6 CRITERIA FOR EXCLUSION FROM EFFICACY ANALYSIS

8.0 LABORATORY EVALUATIONS
8.1 MICROSCOPY
8.2 HAEMOGLOBIN MEASUREMENT
8.3 MOLECULAR STUDIES
8.4 HIGH PERFORMANCE LIQUID CHROMATOGRAPHY (HPLC)

9.0 ADVERSE EVENT MONITORING
9.1 DEFINITIONS
9.2 IDENTIFICATION OF ADVERSE EVENTS
9.3 REPORTING OF ADVERSE EVENTS
9.4 REPORTING OF SERIOUS ADVERSE EVENTS

10.0 STATISTICAL ISSUES
10.1 SAMPLE SIZE
10.2 ANALYTICAL PLAN

11.0 DATA AND SAFETY MONITORING BOARD
11.1 DATA AND SAFETY MONITORING BOARD
11.2 MONITORING PLAN
11.3 STOPPING GUIDELINES

12.0 DATA COLLECTION AND MANAGEMENT
12.1 DATA MANAGEMENT
12.2 DATA QUALITY ASSURANCE AND MONITORING
12.3 RECORDS

13.0 PROTECTION OF HUMAN PARTICIPANTS
13.1 INSTITUTIONAL REVIEW BOARD REVIEW
13.2 INFORMED CONSENT PROCESS
13.3 RISKS AND DISCOMFORTS
13.4 COMPENSATION
13.5 ALTERNATIVES
13.6 CONFIDENTIALITY OF RECORDS

14.0 STUDY TEAM AND PARTICIPATING SITES

15.0 FUNDING AGENCY

16.0 CAPACITY BUILDING

17.0 REFERENCES

APPENDICES

APPENDIX A	SCREENING AND ENROLLMENT
APPENDIX B	INFORMATION SHEET
APPENDIX C	INFORMED CONSENT FORMS
APPENDIX D	INITIAL SCREENING FORM
APPENDIX E	CLINICAL SCREENING FORM
APPENDIX F	ASSENT FORM
APPENDIX G	CASE RECORD FORMS
APPENDIX H	ADVERSE EVENT GRADING SCHEME
APPENDIX I	WEIGHT-BASED TREATMENT GUIDELINES
APPENDIX J	ACCEPTIBILITY QUESTIONNAIRE
APPENDIX K	ADVERSE EVENT REPORTING FORM

STUDY SUMMARY

Title	IPT in schoolchildren: Comparison of the efficacy, safety, and tolerability of antimalarial regimens
**Study design**	Randomized, single-blinded, placebo controlled trial
**Participants and sample size**	Children aged ≥ 8 years attending primary schools<br>The initial target sample size is 760 children (190 per study arm)
**Study site**	The study will be conducted at one of the Uganda Malaria Surveillance Project (UMSP) sentinel sites in Tororo, an area with high transmission intensity
**Selection criteria**	**Inclusion criteria**<br>1. Age ≥ 8 to < 14 years (boys); ≥ 8 to < 12 years (girls)<br>2. Student enrolled at participating school in classes 1 to 7<br>3. Provision of informed consent from parent or guardian<br>4. Provision of assent by student<br>**Exclusion criteria**<br>1. Known allergy or history of adverse reaction to study medications<br>2. Onset of menstruation (girls)<br>3. Fever (≥ 37.5ºC axillary) or history of fever in the previous 24 hours<br>4. Evidence of severe malaria or danger signs<br>5. Ongoing antimalarial treatment<br>6. Haemoglobin < 7.0 g/dL<br>7. Parasite density ≥ 10,000/ul
**Study intervention**	Participants will be randomized to one of four treatment arms and followed for 42-d:<br>1. Sulfadoxine-pyrimethamine (SP)<br>2. Amodiaquine + sulfadoxine-pyrimethamine (AQ+SP)<br>3. Dihydroartemisinin-piperaquine (DP)<br>4. Placebo<br>Participants in the SP arm will also receive placebo tablets on days 1 and 2 to ensure that the number of doses received is identical in the other treatment groups.
**Primary objective**	To compare the efficacy of different combination antimalarial regimens, including AQ+SP, DP, and placebo to SP for IPT in schoolchildren, as measured by risk of parasitaemia (unadjusted by genotyping) after 42 days of follow-up. This will assess both the efficacy for treatment of asymptomatic infections and the efficacy for prevention of new infections.
**Secondary objectives**	1. To compare the efficacy of different antimalarial regimens, including AQ+SP and DP, to SP for treatment of asymptomatic infection, as measured by risk of recurrent parasitaemia (adjusted by genotyping) in children who were parasitaemic at enrollment.<br>2. To compare the efficacy of different antimalarial regimens, including AQ+SP and DP, to SP for prevention of new infections, as measured by risk of recurrent parasitaemia (adjusted by genotyping) in all children.<br>3. To compare the safety and tolerability of SP, AQ+SP, and DP to that of placebo for IPT in schoolchildren, over 42 days of follow-up.<br>4. To evaluate the acceptability of the different IPT regimens by study participants.

LIST OF ABBREVIATIONS AND ACRONYMS

ACT	artemisinin-based combination therapy
ACPR	adequate clinical and parasitological response
AE	adverse event
AL	artemether-lumefantrine
AS	artesunate
AQ	amodiaquine
CF	clinical failure
DOMC	Division of Malaria Control
DP	dihydroartemisinin-piperaquine
DSMB	data safety and monitoring board
GCP	good clinical practices
Hb	haemoglobin
IMCI	integrated management of childhood illnesses
IPT	intermittent preventive treatment
ITN	insecticide treated net
IRB	institutional review board
HIV	human immunodeficiency virus
HPLC	high performance liquid chromatography
LMP	last menstral period
LSHTM	London School of Hygiene & Tropical Medicine
MoH	Ministry of Health (Uganda Government)
MU	Makerere University (Kampala, Uganda)
PF	parasitological failure
SAE	serious adverse event
SP	sulfadoxine-pyrimethamine
UCSF	University of California, San Francisco
UMSP	Uganda Malaria Surveillance Project
UNCST	Uganda National Council of Science and Technology
VCD	Vector Control Division, Ugandan Ministry of Health
WHO	World Health Organization

1.0 BACKGROUND

1.1 Introduction

Malaria remains one of the most serious global health problems.[1] It is estimated that between 400 to 900 million episodes of fever occur yearly in African children, probably about half due to malaria, resulting in over one million deaths.[2,3] In Africa, severe anaemia is a major contributor to malaria-associated death.[4,5] In addition to acute illness, chronic manifestations of malaria, including anaemia, neurocognitive dysfunction, developmental delay, and pregnancy-related complications, contribute substantially to the clinical impact and burden of disease.[6] Despite recent commitments to control malaria in Africa, it appears that malaria-specific mortality is rising, accounting for an increasing proportion of overall childhood morality.[7] Typically, malaria control efforts focus on children under five years (and pregnant women) because they bear the brunt of morbidity and mortality. In endemic areas, risk of clinical disease and death declines throughout childhood due to the gradual acquisition of immunity gained through repeated infection.[8] By adolescence, most malaria infections are asymptomatic, although pregnancy again places women at increased risk. While older children generally suffer less mortality and morbidity, malaria in this age group is not insignificant, and is of substantial importance to education of schoolchildren through reduced school attendance, cognition, learning and school performance.[9]

1.2 Burden of malaria in Uganda

Malaria is endemic in 95% of Uganda, and is the leading cause of morbidity and mortality in the country, accounting for 25-40% of all outpatient visits at health facilities, 20% of hospital admissions, and 9-14% of inpatient deaths (Uganda Ministry of Health, unpublished). In Uganda, and many countries in sub-Saharan Africa, malaria remains one of the leading causes of death amongst children under five years.[10] In addition, a recent information update on malaria in Uganda from the Ministry of Health (2000) reported that malaria morbidity is increasing (25-40% of outparticipant visits in 1992-3, 27-51% in 1998 and 29-50% in 1999).

1.3 Intermittent preventive treatment

Intermittent preventive treatment (IPT), the administration of curative doses of anti-malarial treatment at predefined intervals regardless of infection status, is recommended to reduce malaria in pregnancy.[11] Studies of IPT with sufadoxine-pyrimethamine (SP) have demonstrated that treatment reduces the negative impact of malaria in pregnancy, including placental parasitaemia, maternal anaemia, parasite prevalence, and low birth weight.[12-19] In Uganda, IPT in pregnancy (IPTp) has been adopted as policy, with the recommendation that all pregnant women receive a treatment dose of SP in the second and third trimester (http://www.health.go.ug/mcp/mp.html). Given the benefits of IPT in pregnant women, use of IPT is also being investigated among infants (IPTi) and young children (IPTc), and has been shown to reduce anaemia and clinical malaria episodes.[20-24] Although intermittent treatment of malaria has been shown to be beneficial, the method by which IPT exerts its action is unclear.[25] IPT may treat unrecognized infection in asymptomatic individuals who would typically go untreated, and may also prevent new

infections by providing post-treatment prophylaxis. Prevention of new infections may be the more important factor, particularly in areas of high transmission intensity.[25]

1.4 IPT in schoolchildren

In Africa, children under five and pregnant women are typically considered to be at highest risk of malaria-associated morbidity and mortality. However, older children are also at risk, particularly for the chronic effects of malaria infection.[9] Currently, use of IPT among schoolchildren as a method to improve haemoglobin status and school performance through prevention of malaria and treatment of asymptomatic infection is also being explored. Results from a recent trial of IPT, investigating use of amodiaquine + sulfadoxine-pyrimethamine (AQ+SP) in Kenyan schoolchildren found that thrice yearly IPT (administered once each term) reduced the absolute risk of malaria by 0.36 (95% CI 0.31 to 0.41) and anaemia by 0.07 (95% CI 0.02 to 0.12) (Clarke *et al*, unpublished data). Treatment was also associated with a significant improvement in cognitive performance (Clarke *et al*, unpublished data). These findings hold promise for an effective school-based malaria control strategy.

1.5 Role of the education sector

The potential role of the education sector in malaria control, through prevention and treatment, is gaining attention.[26-28] Recently, the World Health Organization published a report on malaria prevention and control in schools, which highlights the potential role of the education sector in malaria control and supports action on malaria in schools.[29] Although there is interest in expanding malaria control activities to schools, guidelines and policies on how to implement prevention and treatment programmes in practice are limited. To help fill this information gap, the World Bank has recently supported an initiative to strengthen the ability of the education sector to address the impact of malaria on school-aged children, and to provide guidelines for incorporating a school-based malaria response, possibly including IPT, into education projects (Simon Brooker, *personal communication*).

1.6 Choice of drugs for IPT

Although IPT has become an important part of malaria control, the optimal regimen remains unclear.[25] When assessing regimens for IPT, factors to consider include the effectiveness, which incorporates drug efficacy, ease of administration, cost, availability, and acceptability, and safety and tolerability.[30] Determinants of the efficacy of an IPT regimen include the ability to successfully treat unsuspected infection, and the ability to prevent new infections by providing post-treatment prophylaxis.[25] The availability of co-formulated drugs for combination regimens, and the likelihood of proper administration and adherence also contribute to effectiveness. Considering these factors, the ideal IPT regimen would be highly efficacious, long-acting, and easy to administer. However, balancing these factors is challenging, and the benefits of long-acting drugs in preventing reinfection must be weighed against the potential risk of driving drug resistance.[31] Cost, availability, and acceptability are also important, particularly for programme effectiveness, and all factors should be considered when selecting a regimen for IPT.

1.7 IPT regimens

All antimalarial regimens used for treatment of uncomplicated malaria are options for IPT, including older monotherapies and newer combination regimens.

1.7.1 Sulfadoxine-pyrimethamine (SP). Of the available regimens, SP has been most widely studied for IPT. Currently SP is recommended for IPT in pregnant women, and is the only regimen included in an IPT policy in Uganda and many other African countries. SP has several advantages that make it attractive for use in an IPT program in schoolchildren, including low cost, wide availability, simple dosing and relatively long elimination half-life (Table 1) . The fact that SP is administered as a single dose ensures 100% adherence with each treatment, which could have a substantial impact on operational effectiveness of IPT. However, resistance to SP has become widespread in Africa, which could limit the utility of this regimen.[25] Regarding efficacy of SP, although the rate of failure when SP is used for treatment of symptomatic malaria is high, the efficacy of SP for treatment of asymptomatic infections and prevention of new infections is unknown. It is also possible that acquired immunity in older children will complement drug action and may compensate for drug resistance. It is thus possible that SP may retain its efficacy in such a situation, which needs further investigation. The advantages of SP would therefore make it the ideal therapy for use in IPT in schoolchildren, and the efficacy, safety and tolerability of the other regimens will need to be compared to SP.

1.7.2 Amodiaquine + sulfadoxine-pyrimethamine (AQ+SP). AQ alone, and in combination with SP have previously been evaluated for IPT.[22] By adding AQ to SP, efficacy is significantly improved, but the dosing becomes more complex, extending to a three-day treatment.[32] In schoolchildren, AQ+SP for IPT was found to achieve 92% parasitological clearance by day 28 post-treatment in Western Kenya, an area with high levels of SP-resistance (Clarke *et al*, unpublished data).

1.7.3 Artemisinin combination therapies (ACTs). Newer ACTs are also potential options for IPT, including AQ plus artesunate (AQ+AS), artemether-lumefantrine (AL, Coartem), and AS+SP. Generally, ACTs are highly efficacious; however, the very short half-life of artemisinin derivatives offers no post-treatment prophylaxis. Artemisinins are rapidly eliminated leaving the partner drug to act on its own, which is a potential downside for all ACT regimens in IPT.[25] AL has been shown to be highly efficacious, and to prevent more new infections than AQ+AS, [33] but the twice daily dosing of AL is a significant disadvantage for IPT. Both AL and AQ+AS have also been selected as first-line therapy for uncomplicated malaria in newly revised antimalarial policies in most African countries (http://www.who.int/malaria/amdp/amdp_afro.htm), which may dissuade policy-makers from incorporating these regimens into IPT programmes. AS+SP has been investigated for IPT in children in Senegal,[23] however, the high level of SP resistance in much of Africa may also limit use of this regimen. A new co-formulated ACT, dihydroartemisinin-piperaquine (DP), is a very attractive option for IPT. DP is highly efficacious and is dosed once daily. The long terminal half-life of piperaquine provides extended post-treatment prophylaxis, and a study comparing DP to AL for

treatment of uncomplicated malaria in Uganda showed that DP was superior for prevention of new infections in an area of intense transmission (Kamya *et al*, in press).

**Table 1. Possible IPT regimens: Comparison of efficacy for treatment of uncomplicated malaria with 28-day follow-up**					
**Regimen**	**Site and transmission intensity[34]**	**Efficacy: treatment of uncomplicated malaria**		**Post-treatment prophylaxis**	**Ease of administration**
		**Risk of treatment failure**	**Risk of recurrent parasitaemia***	**Terminal half-life in health[25]**	**Dosing schedule**
SP †	Tororo[35]<br>EIR = 562<br>2002-2004	>34%	>88%	sulfadoxine 7 days<br>pyrimethamine 3 days	Single dose
AQ+SP		18%	59%	amodiaquine 1-3 weeks?<br>sulfadoxine 7 days<br>pyrimethamine 3 days	Once daily for three days
AQ+AS		12%	74%	amodiaquine 1-3 weeks?<br>artesunate 1 hour	Co-formulated<br>Once daily for three days
AL	Tororo[33]<br>EIR = 562<br>2004-2005	1%	51%	artemether 1 hour<br>lumefantrine 3-4 days	Co-formulated<br>Twice daily for three days
AS+SP	Kampala [36]<br>EIR < 5<br>2001-2002	18%	29%	artesunate 1 hour<br>sulfadoxine 7 days<br>pyrimethamine 3 days	Once daily for three days
DP ‡	Apac<br>(Kamya, in press)<br>EIR = 1586<br>2006	2%	11%	dihydroartemisinin 1 hour<br>piperaquine 22 days	Co-formulated<br>Once daily for three days

* Including risk of new infections
† Efficacy results for SP extrapolated from data collected for CQ+SP
‡ In this study, ITNs were distributed at the time of enrollment, which likely decreased the risk of new infections.

1.8 Safety and tolerability of IPT regimens

Safety and tolerability of IPT regimens is a key issue. Typically, the safety and tolerability of antimalarial regimens is assessed in clinical trials by evaluating treatment of symptomatic cases, often in very young children. However, antimalarial therapy delivered through IPT programmes will generally be administered to asymptomatic children and pregnant women. Use of poorly tolerated therapy for IPT in asymptomatic children is likely to be less acceptable than use of the same treatment for symptomatic malaria. Older asymptomatic children may be more capable of observing and reporting adverse effects of treatment, enabling fuller documentation and quantification of adverse effects.

All IPT regimens under consideration have been shown to be relatively safe and well-tolerated. However, there are concerns about the lower tolerability of AQ and AQ+SP. In Rwanda, adult participants treated for uncomplicated malaria with AQ or AQ+SP

more commonly reported pruritis and fatigue than those treated with SP alone.[37] An additional study from Rwanda indicated that the risk of adverse events was lower with DP than AQ-containing regimens.[38] In the Kenyan trial of IPT with AQ+SP in schoolchildren, anecdotal reports suggest that this combination was associated with a range of mild adverse events, including nausea, weakness and fatigue, which may influence future adherence and acceptability of the intervention (Clarke, unpublished data). Tolerability of available antimalarials among asymptomatic schoolchildren is therefore a key research question that needs to be addressed prior to undertaking large-scale effectiveness studies of IPT.

2.0 RATIONALE

IPT in pregnancy has become an important component of malaria control in Africa.[39] IPT programmes may also benefit infants and children,[24,40] and delivery of IPT to children in schools provides an opportunity to extend malaria control activities to older children. Studies of the efficacy, safety, and tolerability of antimalarial therapy are typically conducted in symptomatic malaria participants. As a result, the published literature on the efficacy and safety of antimalarial regimens may not be generalisable to asymptomatic individuals, and additional research is essential. In addition, the mechanism of action of IPT is uncertain, and assessment of the impact of IPT on treatment of asymptomatic infection, and prevention of new infections is needed.

Although IPT shows promise as an approach to malaria control in schools, the optimal regimen remains unclear. We propose to compare the efficacy, safety and tolerability of different antimalarial regimens in schoolchildren, anticipating that this study will be a ‘pilot’ for future IPT research. We plan to evaluate SP, AQ+SP, DP and placebo in healthy schoolchildren, regardless of infection status. The efficacy of the combination regimens will be compared to that of SP, and the safety and tolerability of all regimens will be compared to that of placebo. From our experience, assessment of the safety and tolerability of antimalarial treatment in African children is complicated by the overlap between common adverse events, symptoms of malaria, and symptoms of common non-malarial illnesses. The inclusion of the placebo arm will allow us to assess the risk of adverse events with each of the regimens, as compared to no treatment.

3.0 STUDY OBJECTIVES

3.1 Primary objective:

To compare the efficacy of different combination antimalarial regimens, including AQ+SP, DP, and placebo, to SP for IPT in schoolchildren, as measured by risk of parasitaemia (unadjusted by genotyping) after 42 days of follow-up. This will assess both the efficacy for treatment of asymptomatic infections and the efficacy for prevention of new infections.

3.2 Secondary objectives:

1. To compare the efficacy of different antimalarial regimens, including AQ+SP and DP, to SP for treatment of asymptomatic infection, as measured by risk of recurrent parasitaemia (adjusted by genotyping) in children who were parasitaemic at enrollment.

2. To compare the efficacy of different antimalarial regimens, including AQ+SP and DP, to SP for prevention of new infections, as measured by risk of recurrent parasitaemia (adjusted by genotyping) in all children.

3. To compare the safety and tolerability of SP, AQ+SP, and DP to that of placebo for IPT in schoolchildren, over 42 days of follow-up.

4. To evaluate the acceptability of the different IPT regimens by study participants.

4.0 STUDY DESIGN

4.1 Overall study design

This will be a randomized, single-blinded, placebo-controlled trial to evaluate the efficacy, safety and tolerability of antimalarial regimens in healthy schoolchildren. The study will be carried out among children aged ≥ 8 years (to < 14 years for boys, and to < 12 years for girls) attending primary schools in Tororo district. Schools will be selected using convenience sampling with the assistance of the district and the education sector. The target population includes children attending primary schools in Uganda. The accessible population includes the children attending the participating primary schools in classes 1 to 7 in Tororo district. Children who meet the selection criteria for participation in the study will be randomized to treatment with one of the four study regimens and will be followed for 42 days. Repeat evaluations will be performed on days 1, 2, 3, 7, 14, 28, and 42 (and any unscheduled day that a student is ill) and will include assessment for the occurrence of adverse events. Treatment efficacy outcomes will be assessed using revised WHO outcome classification criteria.[41] Acceptability of treatment regimens will be assessed using a questionnaire administered to participating students on day 7.

4.2 Classification of treatment outcome

Response to treatment will be classified according to criteria modified from the 2006 WHO system for classification of outcome following treatment for uncomplicated malaria, and will include clinical failure (CF), parasitological failure (PF), and adequate clinical and parasitological response (ACPR).[41]

Table 2. Classification of treatment outcome
**Clinical failure: Days 0 to 42**
– Development of danger signs or severe malaria on Days 0 to 42 in the presence of parasitaemia*   – Temperature ≥ 37.5°C (axillary), or history of fever in previous 24 hours, on Days 3 to 42 in the presence of parasitaemia*
**Parasitological failure: Days 3 to 42**
– Development of hyperparasitaemia (≥ 10,000/ul) on Days 1 to 42*   – Presence of parasitaemia on Days 4 to 41 and axillary temperature < 37.5°C, without previously meeting any of the criteria of clinical failure   – Presence of parasitaemia on Day 42 and axillary temperature < 37.5°C, without previously meeting any of the criteria of clinical failure*
**Adequate clinical and parasitological response: Day 42**
– Absence of parasitaemia on Day 42 irrespective of temperature without previously meeting any of the criteria for clinical failure or parasitological failure

* Requires rescue antimalarial therapy

For all clinical and parasitological failures, molecular genotyping will be used to distinguish recrudescence from new infection (see section 8.3). In the final analysis, treatment outcomes will be dichotomized based on the following definitions:

- Risk of parasitaemia = CFs + PFs (unadjusted by genotyping)
- Clinical failure = All CFs due to recrudescence (adjusted by genotyping)
- Parasitological failure = All PFs due to recrudescence (adjusted by genotyping)

4.3 Outcome measures

4.3.1 Primary outcome. Risk of parasitaemia (unadjusted by genotyping) after 42 days of follow-up

4.3.2 Secondary outcomes

1. Risk of recrudescence (adjusted by genotyping) in children who were parasitaemic at enrollment, after 42 days of follow-up
2. Risk of new infection (adjusted by genotyping) in all children
3. Risk of clinical failure due to recrudescence (adjusted by genotyping) in children who were parasitaemic at enrollment, after 42 days of follow-up
4. Risk of parasitological failure due to recrudescence (adjusted by genotyping) in children who were parasitaemic at enrollment, after 42 days of follow-up
5. Mean haemoglobin at day 42
6. Mean change in haemoglobin between day 0 to day 42
7. Risk of serious adverse events over 42 days of follow-up
8. Risk of all adverse events after 14 and 42 days of follow-up
9. Acceptability of IPT regimens

5.0 PARTICIPANT SELECTION AND ENROLLMENT

5.1 Study site

The study will be conducted among schoolchildren in Tororo district, an area with high malaria transmission intensity (estimated entomologic inoculation rate of 586 infective bites per person-year).[34] The prevalence of malaria infection among primary schoolchildren in Tororo is 51%, and 19% of children are anaemic (Simon Brooker, *personal communication).* Hookworm is also common, with 42% of children infected. Information on primary schools from West Budama North school district in Nagongera sub-county in Tororo is provided in Table 3.

**Table 3. Primary schools in Nagongera sub-county**		
**School**	**Estimated distance from Nagongera health center**	**Enrollment**
Rock Hill	< 1 km	1110
Nagongera Girls	1.5 km	1151
St. Joseph Nagongera	3 km	787
Mahanga	3 km	532
Maundo	3 km	832
Bishop Yona Okoth Memorial	3 km	1026
Pokongo Rock	4 km	931
Namwaya	2.5 km	892
Okwira	3.5 km	903
Walaweji	3 km	799
Mukwana	3 km	659
Soni Ogwangi	6 km	240
Pagoya	4km	687
Matindi	4km	553
**Total enrollment**		**11,102**

The study will be conducted by the Uganda Malaria Surveillance Project (UMSP) in collaboration with the Uganda Vector Control Division and the London School of Hygiene and Tropical Medicine. UMSP was established in 2001 to enhance local research capacity and expand existing infrastructure with the goal of providing sustainable progress in malaria control in Uganda. UMSP has extensive expertise and experience in collecting "state of the art" drug efficacy data in studies with large sample sizes, extended follow-up, use of molecular genotyping to distinguish recrudescence from new infections, systematic collection of data on drug safety and tolerability, and quality control.

5.2 Selection criteria

Children enrolled in participating schools will be assessed for the following eligibility criteria:

5.2.1 Inclusion criteria

1. Age ≥ 8 to < 14 years (boys); ≥ 8 to < 12 years (girls)
2. Student enrolled at participating school in classes 1 to 7
3. Provision of informed consent from parent or guardian
4. Provision of assent by student

5.2.2 Exclusion criteria:

1. Known allergy or history of adverse reaction to study medications
2. Onset of menstruation (girls)
3. Fever (≥ 37.5°C axillary) or history of fever in the previous 24 hours
4. Evidence of severe malaria or danger signs
5. Ongoing antimalarial treatment
6. Haemoglobin < 7.0 gm/dL
7. Parasite density ≥ 10,000/ul

5.3 Initial recruitment and consent

Schools in Tororo district will be selected using convenience sampling with the assistance of the district and the education sector. Prior to the onset of the study, staff from participating schools will be sensitized about the study and plans for recruitment and follow-up. Group meetings will then be held with the parents/guardians of schoolchildren aged ≥ 8 to < 14 years (boys); ≥ 8 to < 12 years (girls), who are enrolled in classes 1 to 7 (Appendix A). The group meetings will be held at a convenient location within the community. During the meetings, the purpose and procedures of the study will be discussed, an information sheet will be distributed (Appendix B), and written informed consent will be sought from the parents/guardians (section 10.2). Consent to participate in the research study and consent for future use of biological specimens will be sought (Appendix C). Information about all children, including age, gender, any history of known allergies or adverse reactions to study medications, and onset of menstruation in girls will be obtained from parents/guardians and captured on an initial screening form (Appendix D), but only children for whom consent is provided will have a study number assigned, and will undergo further clinical screening at school (Appendix E). Details about the location of the students' homes will also be obtained from the parent/guardians to facilitate tracing in the event of absence from school on subsequent follow-up visits.

5.4 Screening and enrollment of schoolchildren

Further clinical screening will be conducted at the participating schools (Appendix E). Assessment for eligibility will be done by the study physicians, and interviews will be conducted in the appropriate language with the schoolchildren. During the screening process, the study physicians will assess for eligibility criteria (including onset of menstruation in girls) through conversations with the student, and will seek assent from the student to participate in the study (Appendix F). Children meeting these criteria will undergo a history and physical examination, including measurement of temperature. Children will specifically be evaluated for evidence of clinical conditions requiring treatment, including presence of fever or history of fever, or evidence of danger signs or severe malaria (Table 4). In such situations, children will be excluded and treated appropriately. If there is evidence of severe illness, children will be referred for

additional evaluation and treatment. In addition, children currently receiving antimalarial treatment will be excluded.

Table 4. Danger signs / Severe malaria
– Unarousable coma (if after convulsion, > 30 min)
– Repeated convulsions (> 2 within 24 h)
– Recent convulsions (1-2 within 24 h)
– Altered consciousness (confusion, delirium, psychosis, coma)
– Lethargy
– Unable to drink
– Vomiting everything
– Unable to stand/sit due to weakness
– Severe anaemia (Hb < 5.0 gm/dL)
– Respiratory distress (labored breathing at rest)
– Jaundice (yellow colouring of eyes)

Children fulfilling the clinical selection criteria will have a fingerprick blood sample obtained for haemoglobin measurement, for thick and thin blood smear, to save a bloodspot on filter paper for future molecular testing, and to assess for prior antimalarial treatment by high performance liquid chromatography (see section 8.4 for details of laboratory evaluations). Children with a haemoglobin level < 7.0 g/dL will be excluded and treated appropriately. After blood is obtained by fingerprick, the students will be referred to the study nurse for treatment allocation and treatment with the study medications. A standardized assessment will be carried out to document the presence and severity of symptoms present on the day of treatment (Appendix G). This standardized assessment will be used as the baseline for monitoring of any future adverse events (Appendix H and K). On day 0, the child will be given a study identification card, indicating the dates of scheduled follow-up assessments. On day 0, a stool sample will also be collected to assess for the presence and intensity of helminth infections. Results of the Giemsa-stained thick and thin blood smears obtained on day 0 will not be available until after the children have been treated. Enrollment will be finalized on day 1 when the results of the thick blood smears are available. Students will return for evaluation on day 1 and will be excluded from the study, and treated appropriately, if the parasite density is ≥ 10,000/ul.

6.0 STUDY INTERVENTION

6.1 Randomization

Computer generated randomization lists will be created by a member of the project who will not be directly involved in the conduct of the study. Sealed copies of the original randomization lists and documentation of the procedure used to generate the lists will be stored in the project administrative offices in Kampala. Prior to the onset of the study, sealed copies of the randomization lists will be distributed to the study nurse responsible for treatment allocation.

6.2 Treatment assignment and allocation

Participants will be randomly assigned to one of the four treatment arms (SP, AQ+SP, DP, or placebo). Randomization will be done according to the pre-determined randomization list. Treatment assignment and administration of medications will be performed by the study nurse. To allocate participants to the appropriate treatment group, the study nurse will select the next available treatment number and corresponding study regimen. The study nurse will record the date and time of treatment assignment and the participant's study number.

6.3 Study treatments

Table 5. Drug formulation and labeling		
**Regimen**	**Trade name (Manufacturer)**	**Class**
Sulfadoxine-pyrimethamine (500mg/25mg)	Fansidar / Roche	Antifolate combination
Amodiaquine (200mg)	Camoquin / Pfizer (formerly Parke-Davis)	4-aminoquinoline
Dihydroartemisinin-piperaquine (40mg/320mg)	Duocotexcin / Holley-Cotec Pharmaceuticals	Artemisinin derivative + bisquinoline

6.4 Dosing of study drugs

All participants will receive one dose of medication for 3 days. Study medications will be administered according to weight-based guidelines (Appendix I). Dosing of SP is based on the sulfa component, and the dosing of placebo will mimic that of AQ (10 mg/kg daily).

Table 6. Treatment administration			
**Treatment group**	**Day 0**	**Day 1**	**Day 2**
**SP**	SP (25 mg/kg)	Placebo	Placebo
**AQ+SP**	AQ (10 mg/kg)	AQ (10 mg/kg)	AQ (10 mg/kg)
	SP (25 mg/kg sulfa)	—	—
**DP**	DP (2.1/17.1 mg/kg)	DP (2.1/17.1 mg/kg)	DP (2.1/17.1 mg/kg)
**Placebo**	Placebo	Placebo	Placebo

6.5 Blinding

Study medications will not be identical in appearance or taste, but the number of doses received will be similar for children in all treatment groups. Participants will not be informed of their treatment regimen, and all study staff involved in the assessment of participant outcomes, including the study clinicians (responsible for clinical assessment and measurement of temperature) and laboratory technicians (responsible for reading thick blood smears and determining parasite density) will be blinded to the treatment group assignments

6.6 Administration of study drugs

All study drugs will be administered by the study nurses at the schools. Treatment will be directly observed. The study nurse will record the date and time study drugs are administered. Study drugs will be given as tablets or fractions of tablets to be taken orally with a glass of water. The study nurse will directly observe consumption of study drug. Participants will be observed for 30 minutes to ensure that the medications are not vomited. Any participant who vomits the medication within 30 minutes of administration will be retreated with a second dose. Any participant who vomits repeatedly (> 3 times) will be classified as a clinical failure based on evidence of danger signs (Table 2) and will be referred for further evaluation and appropriate treatment.

7.0 FOLLOW-UP EVALUATION AND PROCEDURES

7.1 Scheduled follow-up procedures

All participants will be followed for 42 days. Repeat evaluations will be done at the school on days 1, 2, 3, 7, 14, 28, and 42, and any unscheduled day that a participant is ill, which will involve obtaining blood samples to evaluate efficacy outcomes, and monitoring the occurrence of adverse events. At each repeat visit, temperature will be measured and a focused physical examination will be performed. If the child is febrile (axillary temperature $\geq$ 37.5°C) or gives a history of fever within the past 24 hours, a fingerprick blood sample will be obtained for repeat thick smear, and filter paper sample. Haemoglobin will be re-evaluated on day 42. At each follow-up visit, study clinicians will assess participants according to a standardized clinical record form, to allow objective and complete quantification of adverse events and tolerability (Appendices G and H). Participants who are absent from school on the day of a scheduled visit will be visited at home and, if necessary, transported to school for evaluation by the study physicians.

Table 7. Follow-up Schedule

	Day 0	Day 1	Day 2	Day 3	Day 7	Day 14	Day 28	Day 42	Unscheduled day
Study drugs	X	X	X						
History	X	X	X	X	X	X	X	X	X
Temperature	X	X	X	X	X	X	X	X	X
Physical exam	X	X	X	X	X	X	X	X	X
Blood smear	X			X	X	X	X	X	X
Filter paper sample	X			X	X	X	X	X	X
Haemoglobin	X							X	
Assessment for AEs	X	X	X	X	X	X	X	X	X
Student questionnaire					X				

X = perform this task

7.2 Unscheduled follow-up

If a participant falls ill on a day which no follow-up assessment is scheduled, they will be instructed to inform school staff of their illness (if they are able to attend school). The

school staff will be instructed to notify the study team of the participant's illness so that appropriate follow-up can be arranged. Members of the study team will visit participating schools every weekday during the study period and will ensure appropriate follow-up of any ill participants. If a participant falls ill and is not able to attend school, they will be instructed to attend a designated study clinical site (presenting their study identification card) for evaluation and treatment. Study team members will also visit all designated clinical sites every day (including weekends) to ensure appropriate follow-up of study participants.

7.3 Management of malaria

Rescue therapy with antimalarials will be provided in the following situations (Table 2).

- Development of danger signs or severe malaria on Days 0 to 42 in the presence of parasitaemia
- Development of hyperparasitaemia (≥ 10,000/ul) on Days 1 to 42
- Temperature ≥ 37.5°C (A), or history of fever in previous 24 hours, on Days 3 to 42 in the presence of parasitaemia
- Presence of parasitaemia on Day 42 and axillary temperature < 37.5°C (A), without previously meeting any of the criteria of clinical failure

Any participant who meets requirements for rescue therapy during follow-up and is diagnosed with uncomplicated malaria will be treated with AL. Any participant, who meets requirements for rescue therapy and is diagnosed with severe malaria or danger signs will be referred for treatment with quinine. Any participant who meets requirements for rescue therapy will continue to be followed for the full 42 days.

7.4 Management of non-malaria illnesses

Participants who are found to have illnesses other than malaria will receive standard-of-care treatment from the study physicians, according to standardized algorithms, or will be referred appropriately. We will avoid the routine use of medications with antimalarial activity, including tetracycline, antifolate, and macrolide antibiotics, when acceptable alternatives are available.

7.5 Evaluation of acceptability

Participants will be interviewed on day 7 using a semi-structured questionnaire to capture data on the acceptability of treatment (Appendix J).

7.6 Criteria for exclusion from efficacy analysis

Participants will be excluded from further study participation in the following situations:

1. If consent to participate in the study is withdrawn
2. If a child is lost to follow-up

If any of the following occurs, the participant will continued to be followed for the full 42 days, but will not have an efficacy outcome assigned:

1. Use of antimalarial drugs outside of the study protocol
2. Incomplete treatment with study medications

8.0 LABORATORY EVALUATIONS

8.1 Microscopy

Thick and thin blood smears will be stained with 2% Giemsa for 30 minutes and read by experienced laboratory technologists who are not involved in direct participant care. Parasite densities will be calculated by counting the number of asexual parasites per 200 leukocytes (or per 500 leukocytes, if the count is <10 asexual parasites/200 leukocytes), assuming a leukocyte count of 8,000/µl. A blood smear will be considered negative when the examination of 100 high power fields does not reveal asexual parasites. Gametocytaemia will also be determined from thick smears. Thin smears will be used for parasite species identification. For quality control, all slides will be read by a second microscopist and a third reviewer will settle any discrepant readings.

8.2 Haemoglobin measurement

Haemoglobin will be measured from fingerprick blood samples using a portable spectrophotometer (HemoCue, Anglom, Sweden).

8.3 Molecular studies

Each time a thick blood smear is obtained blood will also be collected onto filter paper. Samples will be collected by fingerprick sampling. Blood will be placed onto filter paper in approximately 25 ul aliquots per blood spot (4 blood spots per sample). The samples will be labeled with study numbers and dates, air-dried, and stored in small, sealed sample bags at ambient temperature with desiccant. Parasite DNA will subsequently be removed from the filter paper and prepared for molecular analysis using a chelex extraction method. Genotyping will be performed on all participants with parasitaemia during follow-up, who had parasitaemia on day 0. Genotyping of parasites collected at baseline (day 0) and the day of recurrent parasitaemia will be done to distinguish between true recrudescence and new infections. Briefly, selected regions of the merozoite surface protein-2 gene, merozoite surface protein-1 gene, and 6 microsatellite markers will be amplified using PCR and characterized based on sequence and size polymorphisms identified by gel electrophoresis.[42] Genotyping patterns on the day of recurrent parasitaemia will be compared with those at treatment initiation using GelCompar II software (Applied Maths). Additional molecular studies may include analyses of polymorphisms in parasite and/or human genes for mutations that may impact on clinical malaria. Molecular studies will be performed only for research purposes and will have no impact on the clinical management of study participants.

8.4 High performance liquid chromatography (HPLC)

Blood samples collected on day 0, and on any other day that a participant is suspected to have taken an antimalarial outside of the study protocol, will be screened to detect the presence of antimalarial drugs or their metabolites by HPLC methods developed at LSHTM (Harparkash Kaur, personal communication).

9.0 ADVERSE EVENT MONITORING

9.1 Definitions

An adverse event (AE) is defined as "any untoward medical occurrence in a participant or clinical investigation participant administered a pharmaceutical product that does not necessarily have a causal relationship with this treatment" (ICH Guidelines E2A). An adverse event can further be broadly defined as any untoward deviation from baseline health which includes (International Centers for Tropical Disease Research Network Investigator Manual, Monitoring and Reporting Adverse Events, 2003):

- Worsening of conditions present at the onset of the study
- Deterioration due to the primary disease
- Intercurrent illness
- Events related or possibly related to concomitant medications

A serious adverse event (SAE) is defined as an experience that results in any of the following outcomes:

- Death during the period of study follow-up
- Life-threatening experience (one that puts a participant at immediate risk of death at the time of the event)
- Inpatient hospitalization during the period of study follow-up
- Persistent or significant disability or incapacity
- Specific medical or surgical intervention to prevent one of the other serious outcomes listed in the definition.

9.2 Identification of adverse events

At each follow-up visit (days 1, 2, 3, 7, 14, 28, 42, and any unscheduled day), study clinicians will assess participants according to a standardized clinical record form (Appendix G). A severity grading scale, based on toxicity grading scales developed by the WHO and the National Institutes of Health, Division of Microbiology and Infectious Diseases, will be used to grade severity of all symptoms, physical exam findings, and haemoglobin results (Appendix H). Any new event, or an event present at baseline that is increasing in severity, will be considered an adverse event.

9.3 Reporting of adverse events

For each possible adverse event identified and graded as moderate, severe or life threatening, an adverse event report form will be completed (Appendix K). The following information will be recorded for all adverse experiences that are reported:

- Description of event
- Date of event onset
- Date event reported
- Maximum severity of the event
- Maximum suspected relationship of the event to study medication
- Is the event serious?
- Initials of the person reporting the event
- Was the event episodic or intermittent in nature?
- Outcome

– Date event resolved

9.4 Reporting of serious adverse events

Guidelines for reporting of serious adverse events provided by the Makerere University Research and Ethics Committee, the Ugandan National Council for Science and Technology, the London School of Hygiene & Tropical Medicine, and the data and safety monitoring board (DSMB) will be followed.

10.0 STATISTICAL ISSUES

10.1 Sample size calculations

Sample size calculations are based on the primary endpoint to compare the risk of parasitaemia after 42-days follow-up in each of the different combination antimalarial regimens, including AQ+SP and DP, and placebo, to SP. The following sample sizes have been calculated for children treated with SP and assume that 50% of children will have a negative blood slide at enrollment, while 50% will be positive. Of those with a negative smear, 50% are assumed to have a positive blood smear during 42-day follow-up, while 80% of those with a positive smear at enrollment are assumed to have a positive blood smear during follow-up. Hence, the assumed risk of parasitaemia after 42 days of follow-up among those receiving SP is 65%. No formal adjustments for multiple comparisons of treatment arms have been made.

10.1.1 Initial sample size calculations based on superiority. Initial sample size calculations were based on providing a 95% probability of detecting a treatment effect when the true difference in the risk of parasitaemia is ≥15%. This value is based on previous studies of efficacy of the different regimens. The null hypothesis is that the true difference in the risk of parasitaemia at 42 days follow up between each of the treatment regimens and SP is zero versus an alternative is that there is a difference. Assuming 80% power and 10% losses to follow-up a total of 760 children (190 per arm) are required.[43]

10.1.2 Secondary sample size calculations based on non-inferiority. Once the initial target sample size is reached, an interim report will be prepared for the data and safety monitoring board (DSMB, section 11.0). If no significant difference in efficacy is detected between SP and the alternative regimens, and a decision is made to continue the study based on results on the interim analysis, recruitment will continue until the secondary sample size is reached. Secondary sample size calculations are based on assessing non-inferiority by a one-sided 97.5% confidence interval for the difference in risk of parasitema after 42 days between SP and each of the antimalarial regimens and placebo. The null hypothesis is that SP is inferior to the treatment regimens by more than 10% versus an alternative hypothesis that it is not. The 10% difference in risks is smaller than that assumed for superiority. The value of 10% was chosen since this is the largest increase in risk that can be judged as clinically acceptable and was chosen based on clinical relevance and previous studies of treatment efficacy. Assuming 80% power and 10% losses to follow-up a total of 1600 children (400 per arm) are required i.e. an additional 840 children.[44]

10.2 Analytical plan

10.2.1 Overview. This section briefly describes the statistical methods to be used; a detailed analytical plan will be independently reviewed by the data and safety monitoring board (section 11.0). Data analysis will be primarily performed by the project epidemiologist and the study statistician. Descriptive statistics will be used to summarize baseline characteristics of study participants. Efficacy and safety data will be evaluated using a modified intention-to-treat analysis and will only include participants who meet all selection criteria. Because final selection criteria are assessed on Day 1 after final reading of the enrollment thick and thin smears, some participants randomized to treatment but not fulfilling selection criteria will be excluded from the modified intention-to-treat analysis. Estimates will be presented with their 95% confidence intervals. For all efficacy outcomes, "survival" curves (i.e. has not experienced outcome of interest by time t) will be examined using Kaplan-Meier and formally compared between treatment regimens. Participants excluded after enrollment will be censored at the time of their last assessment. No formal adjustments for multiple comparisons will be made. Statistical tests will use a two-sided significance level of 5%.

10.2.2 Primary outcome: risk of parasitaemia. Risk of parasitaemia after 42 days of follow-up will be estimated for each treatment regimen using results unadjusted by genotyping, and risk differences calculated. Corresponding 95% confidence intervals and hypothesis testing will be carried out for risk difference. Time to first episode of parasitaemia and time to symptomatic parasitaemia (including parasitaemia associated with evidence of danger signs or severe malaria, fever, or history of fever in previous 24 hours) will be estimated. The analysis will also be stratified by presence vs. absence of parasites and age at enrollment.

10.2.3 Secondary outcomes: risks of recrudescence, new infection, clinical failure, and parasitological failure. Risk of recrudescence and risk of new infection will be estimated using results adjusted by genotyping, and risk differences calculated. Corresponding 95% confidence intervals and hypothesis testing will be carried out for risk difference. Time to first episode of parasitaemia and time to symptomatic parasitaemia will be estimated. For risk of recrudescence using genotyping adjusted outcomes, participants with recurrent parasitaemia due to new infections will be censored. Risks of clinical failure and parasitological failure due to recrudescence (adjusted by genotyping) in participants who are parasitaemic at enrollment will also be estimated as time to event. These analyses will also be stratified by age at enrollment.

10.2.4 Secondary outcomes: safety, tolerability, and acceptability. The risk of adverse events after 14 and 42 days of follow-up and the risk of serious adverse events in the treatment groups (SP, AQ+SP, and DP) will be compared to that in the placebo group. The analysis will also be stratified by presence vs. absence of parasites at enrollment. To evaluate acceptability, a questionnaire will be administered to participants on day 7. The acceptability of the different treatment groups will be compared to that of placebo. Categorical variables will be compared between the treatment groups using chi-square tests or Fisher's exact tests and continuous variables will be compared using t-

tests or non-parametric tests where appropriate. No formal adjustments for multiple comparisons will be made. Statistical tests will use a two-sided significance level of 5%.

11.0 DATA AND SAFETY MONITORING BOARD

11.1 Data and safety monitoring board

A data and safety monitoring board will be assembled in conjunction with the LSHTM, consisting at a minimum of a chairman, a safety monitors, a clinical monitor, and a statistician.

11.2 Monitoring plan

An interim report will be prepared for review by the DSMB when the initial target sample size is reached. A shell report for the interim report will be prepared and presented to the DSMB for approval prior to the interim review. For the interim review, the study statistician will prepare a blinded summary (containing information on study progress and data quality, including participant recruitment, participant follow-up, and protocol adherence). Efficacy data and safety data on serious adverse events will be reported by anonymised drug groups. Only the members of the DSMB will have access to the drug codes and will be able to unblind the data in the interim report. In addition, the clinical monitor will be asked to review any serious adverse events identified during the study. The clinical monitor will prepare a report including an assessment of the causality of the events and the report will be presented to the other members of the DSMB for review.

11.3 Stopping guidelines

Interpretation of results and decisions about discontinuation of the study will be made by the members of the DSMB. Stopping guidelines will be outlined in detail in the DSMB shell report, and will be based on the primary outcome (risk of parasitaemia, unadjusted by genotyping, after 42 days of follow-up comparing the alternative regimens, including AQ+SP and DP, to SP), and the incidence of serious adverse events (for all treatment groups as compared to placebo).

12.0 DATA COLLECTION AND MANAGEMENT

12.1 Data management

All clinical data will be recorded onto standardised case record forms by study clinicians. Laboratory data will be recorded in a laboratory record book by the study laboratory technicians and then transferred to the case record forms by the study clinicians. Data will be transferred from the case record forms into a computerised database (EPI INFO 6.04) by data entry personnel and will be double entered to verify accuracy of entry. Two back-up files of the database will be stored on compact discs after each data entry session. For quality control, check programs will be written into the database to limit the entry of incorrect data and ensure entry of data into required fields.

12.2 Data quality assurance and monitoring

All members of the study team will be educated in the study protocol prior to the onset of the trial. The study clinicians will complete case record forms at each participant visit. These forms will be reviewed by the study coordinator for completeness and accuracy. For quality control of thick blood smear slide readings, expert microscopists who will be blinded to the participant's treatment group will repeat the reading of all slides. All discrepant slide readings will be resolved based on the results of a 3rd reading. Study group meetings will be conducted by the coordinator to assess progress of the study, address any difficulties, and provide performance feedback to the members of the study group. In addition members from the core facility will make regular visits to active study sites as needed.

12.3 Records

Case record forms will be provided for each participant. Participants will be identified by their initials and study identification number on the case record form. Participant names will not be entered into the computerised database. All participant record forms will be kept in individual files in a secure filing cabinet in the study clinic. All corrections will be made on case record forms by striking through the incorrect entry with a single line and entering the correct information adjacent to it. The correction will be initialed and dated by the investigator. Additional records will be kept in the clinical and laboratory record books at the core facility in Kampala. The investigators will allow all requested monitoring visits, audits or reviews.

13.0 PROTECTION OF HUMAN PARTICIPANTS

13.1 Institutional Review Board (IRB) review

This protocol and the informed consent documents, including any additional educational or recruitment material, will be reviewed and approved by all IRBs before the trial begins. Any amendments or modifications to this material will also be reviewed and approved by the IRBs prior to implementation. The IRBs will include:

London School of Hygiene & Tropical Medicine (LSHTM) Ethics Committee

Address: Keppel Street, London, WC1E 7HT, UK
Contact Person: Gemma Howe
Phone Number: +44 (0) 20 7927 2802
Email: Ethics@lshtm.ac.uk

Makerere University, Research and Ethics Committee (MUREC)

Address: Makerere University, Faculty of Medicine, Office of the Dean, PO Box 7072, Kampala, Uganda
Contact Person: Dr. Charles Ibingira
Phone Number: +256 (0) 414-530020
Fax Number: +256 (0) 414-531091

Uganda National Council of Science and Technology (UNCST)

Address: Uganda House, 11th Floor, PO Box 6884, Kampala, Uganda
Contact Person: Dr. Thomas Gordon Egwang
Phone Number: +256 (0) 414-250499
Fax Number: +256 (0) 414-234579

13.2 Informed consent process

Meetings will be held with the parents/guardians of children enrolled in standards 3-7 to describe the purpose of the study, the procedures to be followed, and the risks and benefits of participation. Information sheets and consent forms will be provided to the parents or guardians for their review. The parents or guardians will be asked to sign consent for their child to participate in the research study. If a parent or guardian is unable to read or write, his/her fingerprint will be used in substitute for a signature, and a signature from a witness to the informed consent discussion will be obtained. Parents or guardians will be informed that participation of their child(ren) in the study is completely voluntary and that they may withdraw from the study at any time. Written assent to participate in the study will also be obtained from the student at the time of screening.

13.3 Risks and discomforts

13.3.1 Privacy. Care will be taken to protect the privacy of participants, as described in this protocol. However, there is a risk that others may inadvertently see participants' medical information, and thus their privacy compromised.

13.3.2 Risks of randomization. This will be a randomized trial, and some treatment arms may prove to be more or less efficacious, more or less well tolerated, and/or more or less safe than others. Thus, there is the risk that participants will be randomized to less efficacious, less well tolerated, and/or less safe treatment regimens. Interim analysis is planned to limit the number of participants exposed to any regimen that proves to be less efficacious or less safe. Some children will be randomized to receive placebo, however, the risk of receiving placebo in this study is minimal. This study is designed to evaluate asymptomatic children who generally would not be tested or treated for malaria. Children who are febrile or have a history of fever, or who have evidence of severe malaria or danger signs will be excluded from the study, treated appropriately, and referred if necessary. Children with a parasite density ≥ 10,000/ul at enrollment will be also excluded and treated with AL or quinine as appropriate, in accordance with national guidelines. Children enrolled in the study will be closely monitored during the 42 days of follow-up and any child who develops clinical malaria will be treated with AL or quinine as appropriate. In addition, all children who are parasitaemic on day 42 will be treated, regardless of clinical symptoms.

13.3.3 Fingerprick blood draws. Risks include pain, transient bleeding and soft-tissue infection.

13.3.4 Risk of sulfadoxine-pyrimethamine (SP)

SP has generally been the preferred replacement for CQ for the treatment of uncomplicated malaria in Africa. Although technically a combination regimen, SP is generally considered a single antimalarial agent, as its success depends on the synergistic action of its two component inhibitors of folate synthesis. SP is approved in the USA for the treatment of falciparum malaria and for chemoprophylaxis against malaria in travelers, but it is no longer recommended for this second use due to rare, but serious toxicity. Adverse reactions listed on the SP package insert (Roche, USA) are blood dyscrasias (agranulocytosis, aplastic anaemia, thrombocytopenia), allergic reactions (erythema multiforme and other dermatological conditions), gastrointestinal reactions (glossitis, stomatitis, nausea, emesis, abdominal pain, hepatitis, diarrhea), central nervous system reactions (headache, peripheral neuritis, convulsions, ataxia, hallucinations), respiratory reactions (pulmonary infiltrates), and miscellaneous reactions (fever, chills, nephrosis); based on widespread experience with the drug, all of these reactions appear to be uncommon or rare with short-term therapeutic use. The best-documented severe adverse effects with SP are cutaneous reactions, primarily noted when SP was used for long-term chemoprophylaxis in non-African populations. Reported rates of serious reactions to SP in the UK, with long-term use for chemoprophylaxis, were 1:2100, with 1:4900 serious dermatological reactions and 1:11,100 deaths.[45] Estimated rates of toxicity in the US were 1:5000-8000 severe cutaneous reactions and 1:11,000-25,000 deaths.[46] Clinical experience suggests that risks of severe toxicity are much lower with malaria treatment regimens in Africa. Overall, the risk of severe reactions occurring in developing countries with single-dose SP treatment has been estimated at 0.1 per million.[47]

13.3.5 Risk of amodiaquine (AQ)

AQ has been described as “very well tolerated” for routine use,[48] and it was widely used for chemoprophylaxis against malaria in travelers (with weekly treatment) in the past. However, prophylactic use was discontinued due to rare instances of agranulocytosis, aplastic anaemia, and hepatotoxicity, principally associated with use for malarial chemoprophylaxis in travelers.[48,49] Side effects listed as occasional on the package insert (Pfizer/Parke-Davis, Senegal) are nausea, vomiting, diarrhea, lethargy, agranulocytosis and other blood dyscrasias, hepatitis, and peripheral neuropathy. Reported rates of serious reactions to AQ in the UK were 1:2100 blood dyscrasias, 1:31,000 deaths from blood dyscrasias, and 1:15,650 serious hepatotoxicity.[45] Toxicities with short-term use for treatment are expected to be much lower, although data are limited.[49-53] In a review of 40 published and unpublished clinical trials, no severe or life-threatening adverse event was noted.[49] Considering tolerability in 488 AQ-treated patients, gastrointestinal toxicities and pruritis were most commonly reported, and the incidence of adverse events was similar among patients treated with AQ, CQ, and SP.[49] At our study site in Uganda, no serious toxicities were observed with AQ monotherapy (131 treatments).[52]

13.3.6 Risk of artemisinins

Artemisinin derivatives have now been extensively studied, and they are remarkable for a lack of serious toxicity when used for the treatment of malaria.[54] Considering all artemisinins, 15% (12,463) of the patients enrolled in all published antimalarial drug

trials over the past 50 years have received an artemisinin compound, and there are more trials on these compounds than on any other antimalarials (N. White, unpublished communication). In addition to formal studies, artemisinins have now been widely used, with well over a million treatments, mostly of artesunate (AS), in Southeast Asia. The only serious toxicity which has emerged in detailed prospective clinical evaluations is a low risk of type 1 hypersensitivity reactions (estimated risk 1:2833, 95% CI 1:1362-1:6944).[55] Electrocardiograms and detailed neurological, audiometric, and neurophysiological tests have failed to show any evidence for cardiac or neurological toxicity in humans (see below for more details).[56,57]

Animal studies have led to some concerns over artemisinins, particularly regarding cardiac and neurological effects, and reproductive toxicity. As slight QT prolongation was observed in dogs treated with high doses, detailed electrocardiographic studies have been conducted in humans during treatment for falciparum malaria.[57-59] Taking into account effects of malaria, no significant effects of artemisinins on the QT interval were identified.

The neurological effects of artemisinins have been very extensively studied. In mice, rats, dogs, and monkeys, high dosages of intramuscular artemether and arteether produce an unusual and selective pattern of damage to certain brainstem nuclei, particularly those of the auditory and vestibular systems.[60-68]AS is transformed in vivo to dihydroartemisinin, which is the most neurotoxic of the artemisinin derivatives.[69,70] However, in the animal models, orally administered AS and dihydroartemisinin are considerably less neurotoxic than intramuscular artemether or arteether. Differences in toxicity are explained by differences in pharmacokinetics of different compounds and different routes of administration.[65,67,68,71] Neurotoxicity results from the long-lasting blood concentrations that follow intramuscular injection of the oil-soluble compounds, artemether and arteether. Oral administration of artemether or arteether, which provides much more rapid absorption and elimination than intramuscular dosing, leads to markedly less neurotoxicity in mice, although oral artemether can be made more neurotoxic by giving the drug in small repeated doses to simulate the constant exposure that follows intramuscular injection.[65] Artesunate is much less toxic than arteether in rats when administered intramuscularly [62]or orally.[66,67,69] Importantly, with high dose intramuscular injections of artemether and arteether, clinical assessment of mice was a sensitive indicator of neurotoxicity; no mice with normal clinical exams showed histopathology.[69]

The artemisinin derivatives are remarkably well tolerated in humans. In a clinical safety review of 108 studies including 9,241 patients, no serious adverse events or significant toxicity was reported.[72] In addition, a systematic review of artemisinin derivatives for treating uncomplicated malaria, including 41 studies of 5,240 patients, showed no evidence of harmful effects related to artemisinin derivatives.[32] Clinical studies have shown no convincing evidence for neurotoxicity after treatment with artemisinin derivatives, though neurological effects of acute malaria are common. One letter described ataxia and slurred speech after AS therapy, but these findings were consistent

with the course of severe malaria.[73] To specifically evaluate for potential artemisinin-associated auditory toxicity in humans, van Vugt et al. performed clinical neurological evaluations, audiometry and early latency auditory evoked responses in 79 patients treated with multiple doses of artemether or artesunate and 79 matched controls in Thailand, and no evidence of auditory toxicity was detected.[57] Comparisons of patients who had received multiple courses of artemisinin derivatives with age-matched untreated controls showed no significant differences in clinical, audiometric, or auditory evoked potential measurements.[56,57] Even considering the most worrisome dosing regimen, there is no evidence that clinical use of intramuscular artemether has caused neurotoxicity. In a new report, four independent neuropathologists examined the brains of patients who died after treatment with intramuscular artemether, and there was no evidence for the characteristic pattern of neuropathological change seen in the animal studies.(Ref Hien 2003) These results suggest a wide margin of safety for artemisinins in clinical use, particularly when given orally, particularly for water soluble compounds, and most particularly for the most widely studied water-soluble agent, AS.

13.3.7 Risk of dihydroartemisinin-piperaquine (DP). DP is an artemisinin-containing fixed-combination drug developed in China. Recent randomized clinical trials in Cambodia, Vietnam, and Thailand indicate excellent tolerability and high cure rates against multi-drug resistant falciparum malaria. Artemisinin derivatives such as dihydroartemisinin have been used safely in large numbers of participants with uncomplicated or severe malaria. Piperaquine has been used less widely. In a study of the safety and efficacy of DP in 106 Cambodian children and adults with uncomplicated malaria, adverse events were uncommon (< 5%), mild, short lived, and difficult to distinguish from symptoms of malaria (anorexia, nausea, vomiting, abdominal pain, diarrhea, and dizziness).[74] In a safety evaluation of DP in 62 Cambodian children and adults with malaria, DP was found to be safe and well tolerated with no evidence of clinically significant postural hypotension, QTc prolongation, or propensity for hypoglycemia.[75] In a clinical trial of DP in 166 Vietnamese participants with uncomplicated malaria, 3% of participants reported minor adverse events, mostly transient nausea, which were self limited and resolved with the abatement of fever.[76] In a dose-optimization clinical trial of DP in 487 children and adults from Thailand with uncomplicated malaria, DP was well tolerated, with a low incidence of mild adverse events, which were mainly upper gastrointestinal and were similar to those reported in other studies.[77] In a clinical trial of DP in 331 children and adults from Thailand with uncomplicated malaria, DP was well tolerated, with a low incidence of mild side effects and now serious adverse events felt to be likely related to the study drug.[78] DP is now in routine use in Vietnam with no reports of serious adverse events (although with the acknowledgment that there are limited resources available there for pharmacovigilance).

13.4 Compensation

All antimalarial medication, and the evaluation and treatment for some routine medical problems encountered during follow-up will be provided free of charge. If cases are referred by study staff to a health facility for further assessment, transportation will either be provided by the study team, or the costs of transportation will be borne by the project. Medical care that the participant receives which is unrelated to malaria will remain the

primary responsibility of the participant, parent or guardian, although routine medical problems will generally be managed by the study at no cost to the participant.

13.5 Alternatives

Individuals whose parents or guardians choose not to participate in this study will not be enrolled. Children excluded from the study will still be eligible for standard care of medical problems as they arise at the government health dispensaries or other medical facilities in the UMSP sentinel sites.

13.6 Confidentiality of records

Participants, parents and guardians will be informed that participation in a research study may involve a loss of privacy. All records will be kept as confidential as possible. Participants will be identified primarily by their study number and participant names will not be entered into the computerized database. No individual identities will be used in any reports or publications resulting from the study.

14.0 STUDY TEAM AND PARTICIPATING SITES

14.1 Investigators and collaborators

Sarah Staedke
Role in project: Principal investigator
Clinical Senior Lecturer, London School of Hygiene and Tropical Medicine
Co-investigator, Uganda Malaria Surveillance Project

Sian Clarke
Role in project: Co-investigator
Senior Lecturer, London School of Hygiene and Tropical Medicine

Simon Brooker
Role in project: Co-investigator
Reader, London School of Hygiene and Tropical Medicine
Research Fellow, KEMRI/Wellcome Trust Collaborative Programme, Nairobi, Kenya

Ambrose Talisuna
Role in project: Co-investigator
Co-investigator, Uganda Malaria Surveillance Project

Richard Ndyomugyenyi
Role in project: Co-investigator
Vector Control Division, Ministry of Health

Narcis Kabatereine
Role in project: Collaborator
Vector Control Division, Ministry of Health

Benson Estambale
Role in project: Collaborator
University of Nairobi Institute for Infectious and Tropical Diseases, Kenya

Bonnie McGlone
Role in project: Study statistician
Research fellow, London School of Hygiene and Tropical Medicine

Harparkash Kaur
Role in project: Collaborator
London School of Hygiene and Tropical Medicine

Daniel Chandramohan
Role in project: Collaborator
London School of Hygiene and Tropical Medicine

14.2 Participating sites

Uganda Malaria Surveillance Project (UMSP)
Address: Uganda Malaria Surveillance Project, P.O. Box 7475, Kampala, Uganda
Contact Person: Catherine Tugaineyo
Phone Number: +256 (0) 414-530692
Fax Number: +256 (0) 414-540524
Email: ctugaineyo@yahoo.com

London School of Hygiene & Tropical Medicine (LSHTM)
Address: Keppel Street, London, WC1E 7HT, UK
Contact Person: Susan Sheedy
Phone Number: +44(0) 20 7927 2256
Fax Number: +44(0)20 7637 4314
Email: susan.sheedy@lshtm.ac.uk

Vector Control Division, Ugandan Ministry of Health
Address: VCD, Ministry of Health, PO Box 1661
Contact Person: Dr. Narcis Kabatereine
Phone Number: +256 (0) 772-492078
Email: vcd_sci@utlonline.co.ug

15.0 FUNDING AGENCY

Gates Malaria Partnership
Address: 50 Bedford Square, London, WC1B 3DP, UK
Contact Person: Dr. Amit Bhasin

Phone Number: +44 (0) 207 299 4711
Email: amit.bhasin@lshtm.ac.uk

16.0 CAPACITY BUILDING

The research builds upon existing collaborations between LSHTM and the University of Nairobi and the Division of Malaria Control (DOMC) in Kenya, and between LSHTM and the Vector Control Division (VCD) in Uganda. The structure of existing (solid lines) and proposed (dashed lines) collaborations is presented in the schematic below.

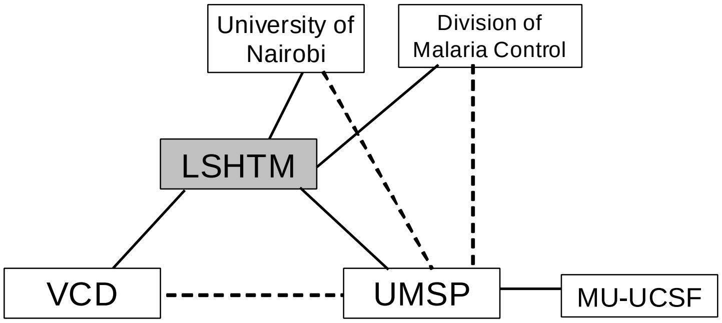

The proposed collaboration with the Uganda Malaria Surveillance Project (UMSP) brings an important scientific dimension to this work, notably expertise in conducting drug efficacy trials. UMSP links academic researchers from the Makerere University - University of California, San Francisco (MU-UCSF) Research Collaboration with the Uganda Ministry of Health, and undertakes malaria research in sentinel sites around Uganda. Current research activities conducted by UMSP and MU-UCSF include antimalarial treatment efficacy studies in contrasting transmission settings, malaria surveillance, pharmacovigilance, and assessment of home-based management of fever, HIV and malaria co-infection, and the utility of rapid diagnostic tests for malaria. Future research directions for UMSP include expansion of surveillance and epidemiological capacity.

This study will contribute to capacity building by strengthening links between the various research organizations, and through training. Staff from VCD in Uganda and from DOMC in Kenya will receive hands-on training in malaria laboratory methods, including an intensive training course in laboratory procedures, developed as part of the ongoing Joint Uganda Malaria Training Programme (JUMP) coordinated by Infectious Diseases Institute and UMSP. In turn, UMSP will gain greater experience in the conduct of community-based clinical trials through collaboration with VCD in Uganda. Hands-on epidemiological training will be provided to UMSP staff by the LSHTM PIs. At least as importantly, the project will help strengthen regional collaboration and skill sharing, and help form the basis for future, regional collaborative research.

17.0 REFERENCES

1. Breman JG, Alilio MS, Mills A. Conquering the intolerable burden of malaria: what's new, what's needed: a summary. *Am J Trop Med Hyg* 2004;**71**(2 Suppl)**:**1-15.
2. Breman J. The ears of the hippopotamus: manifestations, determinants, and estimates of the malaria burden. *Am J Trop Med Hyg* 2001;**64:**1-11.
3. Snow RW, Craig M, Deichmann U, Marsh K. Estimating mortality, morbidity and disability due to malaria among Africa's non-pregnant population [see comments]. *Bull World Health Organ* 1999;**77**(8)**:**624-40.
4. Greenwood BM. The epidemiology of malaria. *Ann Trop Med Parasitol* 1997;**91**(7)**:**763-9.
5. Marsh K, Forster D, Waruiru C, Mwangi I, Winstanley M, Marsh V, Newton C, Winstanley P, Warn P, Peshu N, et al. Indicators of life-threatening malaria in African children. *N Engl J Med* 1995;**332**(21)**:**1399-404.
6. Murphy SC, Breman JG. Gaps in the childhood malaria burden in Africa: cerebral malaria, neurological sequelae, anemia, respiratory distress, hypoglycemia, and complications of pregnancy. *Am J Trop Med Hyg* 2001;**64**(1-2 Suppl)**:**57-67.
7. Snow RW, Trape JF, Marsh K. The past, present and future of childhood malaria mortality in Africa. *Trends Parasitol* 2001;**17**(12)**:**593-7.
8. Marsh K, Snow RW. Host-parasite interaction and morbidity in malaria endemic areas. *Philos Trans R Soc Lond B Biol Sci* 1997;**352**(1359)**:**1385-94.
9. Lalloo DG, Olukoya P, Olliaro P. Malaria in adolescence: burden of disease, consequences, and opportunities for intervention. *Lancet Infect Dis* 2006;**6**(12)**:**780-93.
10. Black RE, Morris SS, Bryce J. Where and why are 10 million children dying every year? *Lancet* 2003;**361**(9376)**:**2226-34.
11. World Health Organization. A strategic framework for malaria prevention and control during pregnancy in the African region. 2004(AFR/MAL/04/01).
12. Schultz LJ, Steketee RW, Macheso A, Kazembe P, Chitsulo L, Wirima JJ. The efficacy of antimalarial regimens containing sulfadoxine-pyrimethamine and/or chloroquine in preventing peripheral and placental Plasmodium falciparum infection among pregnant women in Malawi. *Am J Trop Med Hyg* 1994;**51**(5)**:**515-22.
13. Verhoeff FH, Brabin BJ, Chimsuku L, Kazembe P, Russell WB, Broadhead RL. An evaluation of the effects of intermittent sulfadoxine-pyrimethamine treatment in pregnancy on parasite clearance and risk of low birthweight in rural Malawi. *Ann Trop Med Parasitol* 1998;**92**(2)**:**141-50.
14. Parise ME, Ayisi JG, Nahlen BL, Schultz LJ, Roberts JM, Misore A, Muga R, Oloo AJ, Steketee RW. Efficacy of sulfadoxine-pyrimethamine for prevention of placental malaria in an area of Kenya with a high prevalence of malaria and human immunodeficiency virus infection. *Am J Trop Med Hyg* 1998;**59**(5)**:**813-22.
15. Shulman CE, Dorman EK, Cutts F, Kawuondo K, Bulmer JN, Peshu N, Marsh K. Intermittent sulphadoxine-pyrimethamine to prevent severe anaemia secondary to

malaria in pregnancy: a randomised placebo-controlled trial. *Lancet* 1999;**353**(9153)**:**632-6.
16. Rogerson SJ, Chaluluka E, Kanjala M, Mkundika P, Mhango C, Molyneux ME. Intermittent sulfadoxine-pyrimethamine in pregnancy: effectiveness against malaria morbidity in Blantyre, Malawi, in 1997-99. *Trans R Soc Trop Med Hyg* 2000;**94**(5)**:**549-53.
17. Njagi JK, Magnussen P, Estambale B, Ouma J, Mugo B. Prevention of anaemia in pregnancy using insecticide-treated bednets and sulfadoxine-pyrimethamine in a highly malarious area of Kenya: a randomized controlled trial. *Trans R Soc Trop Med Hyg* 2003;**97**(3)**:**277-82.
18. van Eijk AM, Ayisi JG, ter Kuile FO, Slutsker L, Otieno JA, Misore AO, Odondi JO, Rosen DH, Kager PA, Steketee RW, Nahlen BL. Implementation of intermittent preventive treatment with sulphadoxine-pyrimethamine for control of malaria in pregnancy in Kisumu, western Kenya. *Trop Med Int Health* 2004;**9**(5)**:**630-7.
19. Guyatt HL, Noor AM, Ochola SA, Snow RW. Use of intermittent presumptive treatment and insecticide treated bed nets by pregnant women in four Kenyan districts. *Trop Med Int Health* 2004;**9**(2)**:**255-61.
20. Schellenberg D, Menendez C, Kahigwa E, Aponte J, Vidal J, Tanner M, Mshinda H, Alonso P. Intermittent treatment for malaria and anaemia control at time of routine vaccinations in Tanzanian infants: a randomised, placebo-controlled trial. *Lancet* 2001;**357**(9267)**:**1471-7.
21. Desai MR, Mei JV, Kariuki SK, Wannemuehler KA, Phillips-Howard PA, Nahlen BL, Kager PA, Vulule JM, ter Kuile FO. Randomized, controlled trial of daily iron supplementation and intermittent sulfadoxine-pyrimethamine for the treatment of mild childhood anemia in western Kenya. *J Infect Dis* 2003;**187**(4)**:**658-66.
22. Massaga JJ, Kitua AY, Lemnge MM, Akida JA, Malle LN, Ronn AM, Theander TG, Bygbjerg IC. Effect of intermittent treatment with amodiaquine on anaemia and malarial fevers in infants in Tanzania: a randomised placebo-controlled trial. *Lancet* 2003;**361**(9372)**:**1853-60.
23. Cisse B, Sokhna C, Boulanger D, Milet J, Ba el H, Richardson K, Hallett R, Sutherland C, Simondon K, Simondon F, Alexander N, Gaye O, Targett G, Lines J, Greenwood B, Trape JF. Seasonal intermittent preventive treatment with artesunate and sulfadoxine-pyrimethamine for prevention of malaria in Senegalese children: a randomised, placebo-controlled, double-blind trial. *Lancet* 2006;**367**(9511)**:**659-67.
24. Greenwood B. Review: Intermittent preventive treatment--a new approach to the prevention of malaria in children in areas with seasonal malaria transmission. *Trop Med Int Health* 2006;**11**(7)**:**983-91.
25. White NJ. Intermittent presumptive treatment for malaria. *PLoS Med* 2005;**2**(1)**:**e3.
26. Brooker S, Guyatt H, Omumbo J, Shretta R, Drake L, Ouma J. Situation analysis of malaria in school-aged children in Kenya - what can be done? *Parasitol Today* 2000;**16**(5)**:**183-6.
27. Bundy DA, Lwin S, Osika JS, McLaughlin J, Pannenborg CO. What should schools do about malaria? *Parasitol Today* 2000;**16**(5)**:**181-2.

28. Clarke SE, Brooker S, Njagi JK, Njau E, Estambale B, Muchiri E, Magnussen P. Malaria morbidity among school children living in two areas of contrasting transmission in western Kenya. *Am J Trop Med Hyg* 2004;**71**(6)**:**732-8.
29. World Health Organization. Malaria prevention and control: an important responsibility of a health-promoting school. 2006.
30. Newman RD, Parise ME, Slutsker L, Nahlen B, Steketee RW. Safety, efficacy and determinants of effectiveness of antimalarial drugs during pregnancy: implications for prevention programmes in Plasmodium falciparum-endemic sub-Saharan Africa. *Trop Med Int Health* 2003;**8**(6)**:**488-506.
31. White N. Antimalarial drug resistance and combination chemotherapy. *Philos Trans R Soc Lond B Biol Sci* 1999;**354**(1384)**:**739-49.
32. McIntosh HM. Chloroquine or amodiaquine combined with sulfadoxine-pyrimethamine for treating uncomplicated malaria. *Cochrane Database Syst Rev* 2000;**2**.
33. Bukirwa H, Yeka A, Kamya MR, Talisuna A, Banek K, Bakyaita N, Rwakimari JB, Rosenthal PJ, Wabwire-Mangen F, Dorsey G, Staedke SG. Artemisinin combination therapies for treatment of uncomplicated malaria in Uganda. *PLoS Clin Trials* 2006;**1**(1)**:**e7.
34. Okello PE, W VANB, Byaruhanga AM, Correwyn A, Roelants P, Talisuna A, D'Alessandro U, Coosemans M. Variation in malaria transmission intensity in seven sites throughout Uganda. *Am J Trop Med Hyg* 2006;**75**(2)**:**219-25.
35. Yeka A, Banek K, Bakyaita N, Staedke SG, Kamya MR, Talisuna A, Kironde F, Nsobya SL, Kilian A, Slater M, Reingold A, Rosenthal PJ, Wabwire-Mangen F, Dorsey G. Artemisinin versus nonartemisinin combination therapy for uncomplicated malaria: randomized clinical trials from four sites in Uganda. *PLoS Med* 2005;**2**(7)**:**e190.
36. Dorsey G, Njama D, Kamya MR, Cattamanchi A, Kyabayinze D, Staedke SG, Gasasira A, Rosenthal PJ. Sulfadoxine/pyrimethamine alone or with amodiaquine or artesunate for treatment of uncomplicated malaria: a longitudinal randomised trial. *Lancet* 2002;**360**(9350)**:**2031-8.
37. Fanello CI, Karema C, van Doren W, Rwagacondo CE, D'Alessandro U. Tolerability of amodiaquine and sulphadoxine-pyrimethamine, alone or in combination for the treatment of uncomplicated Plasmodium falciparum malaria in Rwandan adults. *Trop Med Int Health* 2006;**11**(5)**:**589-96.
38. Karema C, Fanello CI, Van Overmeir C, Van Geertruyden JP, van Doren W, Ngamije D, D'Alessandro U. Safety and efficacy of dihydroartemisinin/piperaquine (Artekin((R))) for the treatment of uncomplicated Plasmodium falciparum malaria in Rwandan children. *Trans R Soc Trop Med Hyg* 2006;**100**(12)**:**1105-11.
39. World Health Organization. The Abuja declaration and the plan of action. Geneva, 2003.
40. Egan A, Crawley J, Schellenberg D. Intermittent preventive treatment for malaria control in infants: moving towards evidence-based policy and public health action. *Trop Med Int Health* 2005;**10**(9)**:**815-7.
41. World Health Organization. Guidelines for the treatment of malaria. 2006;**WHO/HTM/MAL/2006.1108**.

42. Greenhouse B, Myrick A, Dokomajilar C, Woo JM, Carlson EJ, Rosenthal PJ, Dorsey G. Validation of microsatellite markers for use in genotyping polyclonal Plasmodium falciparum infections. *Am J Trop Med Hyg* 2006;**75**(5)**:**836-42.
43. Campbell MJ, Julious SA, Altman DG. Estimating sample sizes for binary, ordered categorical, and continuous outcomes in two group comparisons. *Bmj* 1995;**311**(7013)**:**1145-8.
44. Jones B, Jarvis P, Lewis JA, Ebbutt AF. Trials to assess equivalence: the importance of rigorous methods. *Bmj* 1996;**313**(7048)**:**36-9.
45. Phillips-Howard PA, West LJ. Serious adverse drug reactions to pyrimethamine-sulphadoxine, pyrimethamine-dapsone and to amodiaquine in Britain. *Journal of the Royal Society of Medicine* 1990;**83**(2)**:**82-85.
46. Miller K, Lobel H, Satriale R, Kuritsky J, Stern R, Campbell C. Severe cutaneous reactions among American travelers using pyrimethamine- sulfadoxine (Fansidar) for malaria prophylaxis. *Am J Trop Med Hyg* 1986;**35:**451-458.
47. Sturchler D, Mittelholzer ML, Kerr L. How frequent are notified severe cutaneous adverse reactions to Fansidar? *Drug Safety* 1993;**8**(2)**:**160-68.
48. Luzzi GA, Peto TEA. Adverse effects of antimalarials. *Drug Safety* 1993;**8**(4)**:**295-311.
49. Olliaro P, Nevill C, LeBras J, Ringwald P, Mussano P, Garner P, Brausseur P. Systematic review of amodiaquine treatment in uncomplicated malaria. *Lancet* 1996;**348:**1196-1201.
50. Brasseur P, Guiguemde R, Diallo S, Guiyedi V, Kombila M, Ringwald P, Olliaro P. Amodiaquine remains effective for treating uncomplicated malaria in west and central Africa. *Transactions of the Royal Society of Tropical Medicine and Hygiene* 1999;**93**(6)**:**645-50.
51. Gorissen E, Ashruf G, Lamboo M, Bennebroek J, Gikunda S, Mbaruku G, Kager PA. In vivo efficacy study of amodiaquine and sulfadoxine/ pyrimethamine in Kibwezi, Kenya and Kigoma, Tanzania. *Tropical Medicine and International Health* 2000;**5**(6)**:**459-63.
52. Staedke S, Kamya M, Dorsey G, Gasasira A, Ndeezi G, Charlebois E, Rosenthal P. Amodiaquine, sulfadoxine-pyrimethamine, and combination therapy for treatment of uncomplicated falciparum malaria in Kampala, Uganda: a randomized trial. *Lancet* 2001;**358:**368-74.
53. van Dillen J, Custers M, Wensink A, Wouters B, van Voorthuizen T, Voorn W, Khan B, Muller L, Nevill C. A comparison of amodiaquine and sulfadoxine-pyrimethamine as first-line treatment of falciparum malaria in Kenya. *Transactions of the Royal Society of Tropical Medicine and Hygiene* 1999;**93**(2)**:**185-88.
54. Meshnick SR, Taylor TE, Kamchonwongpaisan S. Artemisinin and the antimalarial endoperoxides: from herbal remedy to targeted chemotherapy. *Microbiology Review* 1996;**60**(2)**:**301-15.
55. Leonardi E, Gilvary G, White N, Nosten F. Severe allergic reactions to oral artesunate: a report of two cases. *Trans R Soc Trop Med Hyg* 2001;**95:**182-183.
56. Kissinger E, Hien TT, Hung NT, Nam ND, Tuyen NL, Dinh BV, Mann C, Phu NH, Loc PP, Simpson JA, White NJ, Farrar JJ. Clinical and neurophysiological study

of the effects of multiple doses of artemisinin on brain-stem function in Vietnamese patients. *Am J Trop Med Hyg* 2000;**63**(1-2)**:**48-55.
57. Van Vugt M, Angus BJ, Price RN, Mann C, Simpson JA, Poletto C, Htoo SE, Looareesuwan S, White NJ, Nosten F. A case-control auditory evaluation of patients treated with artemisinin derivatives for multidrug-resistant Plasmodium falciparum malaria. *Am J Trop Med Hyg* 2000;**62**(1)**:**65-69.
58. Price R, van Vugt M, Phaipun L, Luxemburger C, Simpson J, McGready R, ter Kuile F, Kham A, Chongsuphajaisiddhi T, White N, Nosten F. Adverse effects in patients with acute falciparum malaria treated with artemisinin derivatives. *Am J Trop Med Hyg* 1999;**60**(4)**:**547-55.
59. van Vugt M, Ezzet F, Nosten F, Gathmann I, Wilairatana P, Looareesuwan S, White NJ. No evidence of cardiotoxicity during antimalarial treatment with artemether-lumefantrine. *Am J Trop Med Hyg* 1999;**61**(6)**:**964-7.
60. Brewer T, Peggins J, Grate S, Petras J, Levine B, Weina P, Swearengen J, Heiffer M, Schuster B. Neurotoxicity in animals due to arteether and artemether. *Trans R Soc Trop Med Hyg* 1994;**88**(Suppl 1)**:**33-36.
61. Brewer TG, Grate SJ, Peggins JO, Weina PJ, Petras JM, Levine BS, Heiffer MH, Schuster BG. Fatal neurotoxicity of arteether and artemether. *Am J Trop Med Hyg* 1994;**51**(3)**:**251-9.
62. Genovese R, Newman D, Brewer T. Behavioral and neural toxicity of the artemisinin antimalarial, arteether, but not artesunate and artelinate, in rats. *Pharmacol Biochem Behav* 2000;**67**(1)**:**37-44.
63. Genovese RF, Newman DB, Li Q, Peggins JO, Brewer TG. Dose-dependent brainstem neuropathology following repeated arteether administration in rats. *Brain Res Bull* 1998;**45**(2)**:**199-202.
64. Kamchonwongpaisan S, McKeever P, Hossler P, Ziffer H, Meshnick SR. Artemisinin neurotoxicity: neuropathology in rats and mechanistic studies in vitro. *Am J Trop Med Hyg* 1997;**56**(1)**:**7-12.
65. Li QG, Brueckner RP, Peggins JO, Trotman KM, Brewer TG. Arteether toxicokinetics and pharmacokinetics in rats after 25 mg/kg/day single and multiple doses. *Eur J Drug Metab Pharmacokinet* 1999;**24**(3)**:**213-23.
66. Nontprasert A, Nosten-Bertrand M, Pukrittayakamee S, Vanijanonta S, Angus BJ, White N. Assessment of the neurotoxicity of parenteral artemisinin derivatives in mice. *Am J Trop Med Hyg* 1998;**59**(4)**:**519-22.
67. Nontprasert A, Pukrittayakamee S, Nosten-Bertrand M, Vanijanonta S, White N. Studies of the neurotoxicity of oral artemisinin derivatives in mice. *Am J Trop Med Hyg* 2000;**62**(3)**:**409-12.
68. Petras JM, Kyle DE, Gettayacamin M, Young GD, Bauman RA, Webster HK, Corcoran KD, Peggins JO, Vane MA, Brewer TG. Arteether: risks of two-week administration in Macaca mulatta. *Am J Trop Med Hyg* 1997;**56**(4)**:**390-6.
69. Nontprasert A, Pukrittayakamee S, Dondorp AM, Clemens R, Looareesuwan S, White NJ. Neuropathologic toxicity of artemisinin derivatives in a mouse model. *Am J Trop Med Hyg* 2002;**67**(4)**:**423-9.
70. Wesche DL, DeCoster MA, Tortella FC, Brewer TG. Neurotoxicity of artemisinin analogs in vitro. *Antimicrob Agents Chemother* 1994;**38**(8)**:**1813-9.

71. Genovese RF, Newman DB, Gordon KA, Brewer TG. Acute high dose arteether toxicity in rats. *Neurotoxicology* 1999;**20**(5)**:**851-9.
72. Riberio IR, Olliaro PL. Safety of artemisinin and its derivatives. *Med Trop* 1998;**58:**50-53.
73. Miller L, Panosian C. Ataxia and slurred speech after artesunate treatment for falciparum malaria. *New England Journal of Medicine* 1997;**336:**1328-29.
74. Denis MB, Davis TM, Hewitt S, Incardona S, Nimol K, Fandeur T, Poravuth Y, Lim C, Socheat D. Efficacy and safety of dihydroartemisinin-piperaquine (Artekin) in Cambodian children and adults with uncomplicated falciparum malaria. *Clin Infect Dis* 2002;**35**(12)**:**1469-76.
75. Karunajeewa H, Lim C, Hung TY, Ilett KF, Denis MB, Socheat D, Davis TM. Safety evaluation of fixed combination piperaquine plus dihydroartemisinin (Artekin) in Cambodian children and adults with malaria. *Br J Clin Pharmacol* 2004;**57**(1)**:**93-9.
76. Tran TH, Dolecek C, Pham PM, Nguyen TD, Nguyen TT, Le HT, Dong TH, Tran TT, Stepniewska K, White NJ, Farrar J. Dihydroartemisinin-piperaquine against multidrug-resistant Plasmodium falciparum malaria in Vietnam: randomised clinical trial. *Lancet* 2004;**363**(9402)**:**18-22.
77. Ashley EA, Krudsood S, Phaiphun L, Srivilairit S, McGready R, Leowattana W, Hutagalung R, Wilairatana P, Brockman A, Looareesuwan S, Nosten F, White NJ. Randomized, controlled dose-optimization studies of dihydroartemisinin-piperaquine for the treatment of uncomplicated multidrug-resistant falciparum malaria in Thailand. *J Infect Dis* 2004;**190**(10)**:**1773-82.
78. Ashley EA, McGready R, Hutagalung R, Phaiphun L, Slight T, Proux S, Thwai KL, Barends M, Looareesuwan S, White NJ, Nosten F. A randomized, controlled study of a simple, once-daily regimen of dihydroartemisinin-piperaquine for the treatment of uncomplicated, multidrug-resistant falciparum malaria. *Clin Infect Dis* 2005;**41**(4)**:**425-32.

APPENDIX A: SCREENING AND ENROLLMENT

Sensitization and education about the study
Meetings with school staff and parents/guardians of participating schools
Information sheets will be distributed and written informed consent will be sought from parents/guardians of children aged ≥ 8 years, enrolled in classes 1-7

Informed consent
Children for whom written informed consent is provided by their parent/guardian will be assigned a study number and will undergo further screening

Screening at participating schools
Children will be screened for the following selection criteria:
No onset of menstruation (in girls), provision of assent

Clinical and laboratory screening
Children who meet the above criteria will be screened for additional criteria:
Temperature < 38.0°C, no history of fever in past 24 hours,
No evidence of severe malaria or danger signs, Hb ≥ 7.0 g/dL, pd < 10,000/ul

Enrollment and randomization
If all selection criteria are met, child will be enrolled, and randomized to treatment with one of four study treatments (SP, AQ+SP, DP, and placebo)

Treatment and blinding
Directly observed therapy administered by study nurses on days 0, 1, and 2.
All study personnel (except study nurses), participating children, their parents/guardians, and school staff will be blinded.

THE REPUBLIC OF UGANDA

APPENDIX B. INFORMATION SHEET

Study Title: IPT in schoolchildren: Comparison of the efficacy, safety, and tolerability of antimalarial regimens

Introduction

The Uganda Malaria Surveillance Project, the London School of Hygiene and Tropical Medicine, and the Vector Control Division of the Uganda Ministry of Health are doing a research study. We would like to see how well different malaria medicines work to treat and prevent malaria in schoolchildren. To do this, we are carrying out a research study in at least 760 children aged 8 to < 14 years (boys) and 8 to < 12 years (girls) attending primary schools in Nagongera sub-county in Tororo, Uganda. From this study we will learn more about how to control malaria.

The study staff, Dr. Sarah Staedke and members of the Uganda Malaria Surveillance Project and the Vector Control Division, will explain this study to you. Research studies include only people who choose to take part. Please take your time to make your decision about letting your child take part, and discuss your decision with others if you wish. If you have any questions, you may ask the researchers.

Why is this study being done?

Malaria is one of the most important health problems in Uganda. Illness from malaria may prevent children from attending school. Children can also be infected with malaria without feeling sick. Malaria infection of any kind may impact on a child's health and their performance at school. We would like to know more about how to prevent and treat malaria in schoolchildren. We would like to know which malaria medicines work the best. We would also like to know which malaria medicines are tolerated the best, and cause the fewest side effects.

What will happen if I agree to let my child take part in this study today?

If you agree to let your child (or children) take part in this study, we will ask you to give us directions to your house, and will make plans to see your child at his/her school. At the school, the study doctors will talk to your child, and will ask your child if he/she agrees to take part in the study. The study doctors will also examine your child and take a blood sample by fingerprick to examine for malaria parasites, and to measure the blood level. If your child meets all of the criteria for entry into the study, they will be enrolled. The study doctors may exclude your child from the study for the following reasons:

- If your child (girls only) has started to menstruate
- If your child refuses to take part in the study
- If your child has fever or tells the study doctors that they have had fever in the past 24 hours
- If your child has severe malaria or danger signs
- If your child is currently receiving antimalarial treatment
- If your child has severe anemia with a hemoglobin of < 7.0 g/dL
- If your child has malaria with a high parasite count (≥ 10,000/ul)

What will happen if my child is enrolled in this study?

If your child (or children) is enrolled into the study, they will be treated with sulfadoxine-pyrimethamine (Fansidar), or amodiaquine (Camoquin) plus sulfadoxine-pyrimethamine (Fansidar), or dihydroartemisinin-

piperaquine (Duocotecxin), or placebo. A placebo is a pill that does not have medicine inside of it. Treatment will be given at school for three days. The treatment that your child will receive will be determined by a lottery. The chance of being placed into each of the treatment groups is the same. You will not be told which treatment your child has been assigned to receive.

Your child will be followed for a total of 42 days to see how well the medicines work to treat and prevent malaria. The following will take place:
- The study doctors will examine your child at his/her school at least 8 times so we can administer the treatments, judge how well the treatments have worked, and see how well your child has tolerated the medicines.
- Blood samples will be collected on at least 6 days. Approximately 6 drops of blood will be taken by fingerprick to examine for malaria parasites, to measure the blood count, and to store blood samples on paper for future laboratory tests that will not impact on the health care of your child.
- A stool sample will be collected to measure for worm infections.
- If your child misses an appointment, the home health visitor will visit you at your home to find out why your child missed the appointment and to arrange follow-up for your child.
- If, at any time, the treatment given to your child does not seem to be working well, it will be changed to Coartem or quinine. Coartem is the government recommended therapy for treatment of malaria, and quinine is recommended for patients with severe malaria. Your child may develop malaria that is severe even after receiving treatment with study medications. If your child shows any evidence of severe malaria (including persistent vomiting, low blood counts, shaking or fits, confusion, or you cannot wake your child) your child will be referred for possible admission to hospital.
- If your child gets sick at any time while they are taking part in the study, he/she should let the teachers and the study team know. You should seek care for your child from the study team at school, or go to Nagongera Health Center or hospital, if school is not in session.

How long will my child be in the study?

You are being asked to allow your child to take part in this study for up to 42 days or until such a time as you or the study doctors decide that your child should no longer take part in the study. The study doctors may withdraw your child from the study for the following reasons:
- If you chose to withdraw your consent to have your child take part in the study
- If we are unable to locate your child for both doses of study medications, or for follow-up appointments

What risks can I expect if my child takes part in the study?

- Which treatment your child is given will be determined by chance. The treatment your child receives may prove to be less effective or have more side effects than the other study treatment or than other available treatments. This will not be known until after the study is completed.
- Serious health problems have rarely been reported following treatment with the study medications. Sulfadoxine-pyrimethamine (Fansidar) – itching, rash, gastrointestinal reactions (stomach upset), headache, convulsions, dizziness, weakness, malaise (general unwell feeling); Amodiaquine (Camoquin) – nausea, vomiting, diarrhea, lethargy (tiredness); Dihydoartemisinin-piperaquine (Duocotecxin) – nausea, diarrhea, vomiting, abdominal pain, anorexia, itching, rashes, and dizziness may occur occasionally (1-10% of the time) in patients.
- Your child will be monitored closely after receiving treatment for malaria with the study medications for any possible side effects of the drugs and will receive appropriate medical care for any such problem during the course of the study. If your child develops severe shaking or fits, is having trouble breathing, cannot eat, drink, or breast feed, or cannot be woken from sleep, they should be brought to the clinic or hospital as soon is possible.

- The risks of drawing blood from a fingerprick include temporary discomfort, bruising, skin infection, and fainting. The amount of blood removed will be too small to affect your child's health.
- The research treatments may have side effects that no one knows about yet. The researchers will let you know if they learn anything that might make you change your mind about your child's participation in the study.

Are there benefits if my child takes part in the study?

- The potential benefit to your child is that the treatment received may prove to be more effective than the other study treatments or than other available treatments, although this cannot be guaranteed.
- Your child will receive clinical care from the medical officers and nurses of the study staff. This will include care for unscheduled sick visits.
- The knowledge gained from this study will help the country of Uganda in determining the best way to treat and prevent malaria in schoolchildren.

What other choices do I have if I do not allow my child take part in this study?

You are free to choose not to let your child take part in the study. If you decide not to take part in this study, there will be no penalty to you.

Will information about me and my child be kept private?

All information gathered will be treated as private by the study personnel, and records will be kept securely in locked filing cabinets and offices. No personal identification information such as names will be used in any reports arising out of this research. We will do our best to make sure that the personal information gathered for this study is kept private. However, any study monitors assigned to this study may look at the information you provide. If information from this study is published or presented at scientific meetings, your name and other personal information will not be used.

Who pays for this study?

The Gates Malaria Partnership has funded this study.

What are the costs of taking part in this study? Will I be paid for taking part in this study?

There are no costs to you for taking part in this study. You will not be paid for taking part in this study.

Can I stop my child from being in the study?

You can decide to withdraw your child from the study at any time. Just tell the study researcher right away if you wish your child to stop being in the study.

What are my rights if my child takes part in this study?

Taking part in this study is your choice. You may choose for your child to either to take part or not to take part in the study. If you decide for your child to take part in this study, you may change your mind at any time. No matter what decision you take, there will be no penalty to you in any way.

Who can answer my questions about the study?

You can talk to the researchers about any questions or concerns you have about this study. Contact Dr. Sarah Staedke or other members of the Uganda Malaria Surveillance Project on telephone number 0414-530692. If you have any questions, comments or concerns about taking part in this study, first talk to the researchers. If for any reason you do not wish to do this, or you still have concerns about doing so, you may contact the Dr Elly Katabira, Makerere University Research and Ethical Committee at telephone number 0414-530020.

APPENDIX C: RESEARCH PARTICIPANT INFORMED CONSENT FORM

**Protocol Title:**	**IPT in school children: Comparison of the efficacy, safety, and tolerability of antimalarial regimens**
**Site of Research:**	Tororo, Uganda
**Principal Investigator**:	Sarah Staedke
**Date:**	17 March 2008

I, being of 18 years or older and having full capacity to consent for the children named below, have been informed about this study. The nature, duration, purpose, voluntary nature and inconveniences or hazards that may reasonably be expected have been fully explained to me. I have understood the information regarding the study, and what will happen. I have been given the opportunity to ask questions concerning this study, and these (if any) have been answered to my satisfaction.

I understand that I may at any time during the study, withdraw my consent and withdraw the subject, without any loss or penalty. My refusal to participate will involve no penalty or loss of benefits to which my family are otherwise entitled.

Mark one box with X:

I DO CONSENT: ☐ **I hereby agree to allow my child/children take part in this study**

I DO <u>NOT</u> CONSENT: ☐ **I do not wish my child/children to participate in this study**

Parent/Guardian's name				
Parent/Guardian's Signature or Mark		Date:		
Village				
Identity card number				
List names of all children	Child's name<br>1.	Class	Date of birth	Age
	2.			
	3.			
	4.			

<u>Witness</u>: I hereby confirm that the study has been explained to the parent/guardian. All questions (if any) have also been answered to his/her satisfaction, and he/she has, of his own free will, consented for his child/children to take part in the study.

Name of Witness:			
Signature of Witness:		Date:	

Name of person explaining study:			
Signature:		Date:	
School:			

APPENDIX C: FORM MANYUTHO YEYO GI ‘NGEYO IGIMA IMENYO TIENDE

**Nyinge Maradier:**	**IPT inyithidho masikulu: ki piima tich payBero, koodi tich payath gi chik mere**
**Kama lebedoiye:**	Tororo, Uganda
**Jadwar tiende madwong:**	Sarah Staedke
**Ka:**	17 March 2008

An, bedo angata oro 18 kosa loyo koodi bedo gi meni jie mayeyo ri nyithindho mundiki nyingin piny ka, owachi rani kwongi kisoma me. Ngeri,hongo,atonga, ngeri mamiyiroki koodi chandiroki kosa gima kin’gere ma inyalo ngitcho kwonge bedo ochowi tito rani. Atyeko niagi wachi ma makere gi kisoma, gi gima latimere. Omiyani silwanyi penjo penji ma mako kwongi kisoma, aka gime (kinen nitye) ochowi dwoko muniangani.

Aniag ni anyalo sawa moro jie ihongo makisoma, wiro paro parani gi weyo kiri wach no, mungoye gima arwenyo kosa girachula. Kwero parani bedo lengoye iye girachula kosa keng limi moro mapecho parani oyido ripo limo.

Kethi ranyuthi isanduku achieli gi chali me X:

**AYERE:**	☐	**Ayere gi weyo nyathparani/nyithindho bedo ikisoma me**
**AKIYERE:**	☐	**Akiyere nyathparani/nyithidho bedo ikisoma me**

Nyigi Janu’ol/jakur				
Chingi janu’ol/jakur		Ndelo dwe:		
Chalo				
Namba Ma adentikadi				
Ndiko Ma nyingi nyithindho jie	Nyingi nyathi<br>1.	Kilas	Ndelo munyu’ol	Oro
	2.			
	3.			
	4.			

Mujulisi: Aridho ni kisoma me otiti rijanyu’ol/jakur.Penji jie kanitye ochowi dwoko muniango go, aka go, kwong mito pere, oyeyo nyithindho/nyathi pere bedo ikisoma me.

Nyingi mujulisi:			
Chingi mujulisi:		Ndelo dwe:	

Nyingi ngata tito kisoma:			
Chingi:		Ndelo dwe:	
Sikulu:			

APPENDIX C: INFORMED CONSENT FOR FUTURE USE OF BIOLOGICAL SPECIMENS

**Protocol Title:**	**IPT in school children: Comparison of the efficacy, safety, and tolerability of antimalarial regimens**
**Site of Research:**	Tororo, Uganda
**Principal Investigator**:	Sarah Staedke
**Date:**	17 March 2008

INTRODUCTION

While your child is in this study, blood samples may be taken that may be useful for future research. These samples will be stored long-term at Makerere University Medical School, the London School of Hygiene and Tropical Medicine, and the University of California, San Francisco. Samples may also be shared with investigators at other institutions.

WHAT SAMPLES WILL BE USED FOR

Your child's blood and the malaria parasites in it will be used to study malaria and the response of this disease to treatment. Results of these studies will not affect your child's care.

1. These samples will be used for future research to learn more about malaria and other diseases.
2. Your child's samples will be used only for research and will not be sold or used for the production of commercial products.
3. Genetic research may be performed on samples. However, no genetic information obtained from this research will be placed in your child's medical records. These samples will be identified only by codes so that they cannot be readily identified with your child.
4. For any future genetic studies done on your child's samples not related to the current study, permission will first be sought from the appropriate committee, including the Makerere University Research and Ethics Committee Institutional Review Board, the London School of Hygiene and Tropical Medicine Ethics Committee, or the University of California, San Francisco Committee on Human Research.

LEVEL OF IDENTIFICATION

Your child's samples will be coded so that the child's name cannot be readily identified. Reports about research done with the samples will not be put in the medical record and will be kept confidential to the best of our ability.

In the future, researchers studying your child's samples may need to know more about your child, such as information about age and gender. If this information is already available because of your child's participation in a study, it may be provided to the researcher. Your child's name or anything that might identify you/them personally will not be provided. You will not be asked to provide additional consent.

RISKS

There are few risks to your child from future use of the samples. A potential risk might be the release of information from your child's health or study records. Reports about research done with your child's samples will not be put in the health record, but will be kept with the study records. The study records will be kept confidential as far as possible.

BENEFITS

There will be no direct benefit to your child. From studying your child's samples we may learn more about malaria or other diseases: how to prevent them, how to treat them, how to cure them.

RESEARCH RESULTS/MEDICAL RECORDS

1. Results from future research using your child's samples may be presented in publications and meetings but patient names will not be identified.
2. Reports from future research done with your child's samples will not be given to you or the doctor. These reports will not be put in your child's medical record.

QUESTIONS

The future use of your child's specimens has been explained to you by the person who signed below and your questions were answered. If you have any other questions about the information here, you may call Dr. Sarah Staedke (0414-530692) at the MU-UCSF Research Collaboration offices.

FREEDOM TO REFUSE

You can change your mind at any time about allowing your child's samples to be used for future research. If you do, contact Dr. Sarah Staedke (0414-530692) at the MU-UCSF Research Collaboration offices. Then your child's samples will no longer be made available for research and will be destroyed. Whether or not you allow us to use your child's samples in future research will not have any effect on your child's participation in this study or future participation in other studies.

WHAT YOUR SIGNATURE OR THUMBPRINT MEANS

Your signature or thumbprint below means that you understand the information given to you in this consent form about your child's specimens and cultures to be used for future research. If you wish to allow your child's specimens and cultures to be used for future research, you should sign or thumbprint below.

Mark one box with X:

I DO CONSENT: ☐ **I hereby agree to allow my child's/children's specimens and cultures to be used for future research**

I DO <u>NOT</u> CONSENT: ☐ **I do not wish my child's/children's specimens and cultures to be used for future research**

Parent/Guardian's name				
Parent/Guardian's Signature or Mark		Date:		
Village				
Identity card number				
List names of all children	Child's name<br>1.	Class	Date of birth	Age
	2.			
	3.			
	4.			

Witness: I hereby confirm that the study has been explained to the parent/guardian. All questions (if any) have also been answered to his/her satisfaction, and he/she has, of his own free will, consented for his child/children's specimens and cultures to be used for future research.

Name of Witness:			
Signature of Witness:		Date:	

Name of person explaining study:			
Signature:		Date:	
School:			

APPENDIX C: NG'EYO IYEYO MAYUWANGE ILA ORO MA FONJIROK KWONGE PAKA GIRANENA WOKI KWONG WADI

**Nyinge Maradier:**	**IPT inyithidho masikulu: ki piima tich payBero, koodi tich payath gi chik mere**
**Kama lebedoiye:**	Tororo, Uganda
**Jadwar tiende madwong:**	Sarah Staedke
**Ka:**	17 March 2008

CHAKIROK MERE

Ka nyathi perin nitye ikisoma me, ilakwanyo remo minyalo medo menyotiende yuwange.Remo mukwanyi me ilakano mahongo mathothi iodi kisoma mukadhomalo ma makerere, sikulu ma lonyo giyath matwoo kama piny liethye ma London, koodi ka kisoma mukadhomalo ma California,San Francisco. Remo mukwanyi me bende inyalo lewo gi juma bende dwaro tiend twoo ma kakisoma mani-man.

GIMA OKWANYI NO NGERI MILE ORO GINE

Remo panyathi perin kudin makelo musuja manitye iye ile oro ma kisoma kwong musuja gi 'ngeri ma twoo me bedo gine ka omiyi yath/konyi.Gima owoki kwong kisoma me kila nyeko miyo nyathiperin konyi.

1. Gima okwanyi ma kisoma kwonge me ile oro mafonjirok kwonge yumalo ma niang kwong musuja gitwoo mani-manijie.
2. Gima okwanyi kwongi nyathiperin me ila oro nyaka ma menyo tiende gi fonjirok Kwonge to kila kitana kosa oro matimo gimorojie makelo pesa.
3. Wanyolo menyo tiendi pek gi dongo maremo woki kwongi gima okwanyi. Too, ongoye wach ma kula kosa kite mu'pondo manitye inyathi perin maile ketho ibaliwa pere ma limo yath/kony. Gima okwanyi. Me ile ketho kwonge namba nyaka ma ngeyo gine ma chero kwako ngeyoni kole a'gima okanyi kwongi nyath perin mu'ngoye kigana-gana.
4. Ikisoma kosa medo menyo kite mupondo yuwange igima okwanyi kwong nyathi. Perin makichale gima isoma pama, meni kutho ile kwanyo bongi komiti, kanya'chiel gi komiti ma Makerere kakisoma mukadhomalo imeyo tiendi twoo koodikitemaraluwa, komiti ma sikulu malonyo gi yeni makama pinyi liethye ma London, kosa komiti ma California ka kisoma ma malo,San Francisco imenyo tiendi jii.

NGERI MA'GEYO

Wale ketho namba igima okwanyi kwong nyathperin magengo kwako ngeyo nyingi nyath perin. Wakila ketho gima owoki kwongi fonjirok kwong gima okwany kwong nyayhi perin ibaliwa pere ma madho yathi/limo kony aka ilekano paka nyaling-ling gi kama wanyalo gine jie.

Jumenyi tiendi twoo ma soma kwong gima okwany kwong nyathi perin yumalo nyalo mito ngeyo mathothi kwong nyathi perin, Nger wachino nyalo bedo paka oro, koodi nyathi manedi [nyako kosa jachwo]. Wanyalo miyojo ngeyo

iwachino ka nitye woki kwong bedo panyath perin ikisoma me. Wakila miyo jo nyingi nyathiperin kosa gimorojie manyalo mio ngitcho/ngeyo ini/ nyathindho no. Wakila doko mito ni ikethi chingin iformi manyutho yeyo man.

JWANGIROK

Juwangirok nitye manoki rinyathiperin aka meno oro nyaka gime okwanyi kwonge yumalo. Juwangirok mutire nyalo bedo wodho wachi kwong kwoo panyathiperin kosa kopi ma ikano ma kisoma. Wakile ketho radwok ma woki kwong fonjirok mu'otim kwong gima okwany kwong nyathiperin ibaliwa ma okan matucho nyaling-ling makwo pere. Wale kano radwoki me kanya'chiel gi kopi magima owoki kwong kisoma pakanyaling-ling aka maber paka wanyalo.

LIMI

Nyathiperin Kila limo mutire kwongi fonjirok me. Woki kwong kisoma kwong gima okwany kwongi nyathiperin, wanyalo fonjere mathothi kwong musuja kosa twoo mani-manjie:Wanyalo fonjere ngeri magen'go jo,miyokonyi, gibotho jo.

RADWOK MAGIMA IMENYO TIENDE /KOPI MA MAKO KWONG KWO

1. Wanyalo wodho yuwange gima owoki kwong medo oro /fonjirok kwong gima okwa nyi kwong nyathi perin ipapula kodi iromo to nyigi jatwoo kila'ngeyo.
2. Radwok kwong fonjirok ma yuwange mutim kwong gima okwanyi kwong nyathi perin kilamiyin kosa musawo pere. Wakila ketho radwok me ifilo kosa kopi malimo konyi panyathiperin.

PENJI

Oro gima okwanyi kwong nyath perin yuwange ngata oketho nyinge pinyi ka ochowo tito rini aka penji perin odwok. Ka initye gipenji mani-man jie mamako kwong wachi manitye ka,inyalo lwongo musawo Sarah Staedke inamba me (0414-530692) I MU-UCSF offisi ma gima'menyo tiende romo'iye.

INIGI THWOLO MA KWERO

Initye gi thwolo ma wiro paro perin kwong oro gimo'okwanyi kwongi nyath perin ma fonjiroki kwonge yuwange sawa morojie.Ka iwiro paro perin,romi gi musawo Sarah Staedke isimo me (0414-530692) I MU-UCSF offisi ma gima imenyo tiende romo'iye.Go le temo swa ni gima okwanyi kwong nyathiperin onyek woko maku'odoko odong ma menyo tiende yuwange. Kada iyeyo kosa ikiyeyo wan oro gima okwany kwong nyath perin yuwange kila nyeko bedo panyath perin ikisoma me kosa mabino yumalo.

GIMA CHINGIN MIKETHO / IFUYO NYUTHO

Chingin miketho kosa ifuyo pinyi ka nyutho ni iniang wach mumiyin ka iformi manyutho yerok me mamako kwong gime okwanyi mara'ora kwong nyathi perin gi thene-thene mere mafonjirok yumalo. Ka imto yeyo gima okwanyi kwong nyath perin githene-thene mere 'ori ma medo fonjirok kwonge yuwange, iripo ketho / fuyo chingin piny ka.

Kethi ranyuthi isanduku achieli gi chali me X:

**AYERE:**	☐	**I hereby agree to allow my child's/children's specimens and cultures to be used for future research**
**AKIYERE:**	☐	**I do not wish my child's/children's specimens and cultures to be used for future research**

Nyigi Janu'ol/jakur				
Chingi janu'ol/jakur		Ndelo dwe:		
Chalo				
Namba Ma adentikadi				
Ndiko Ma nyingi nyithindho jie	Nyingi nyathi<br>1.	Kilas	Ndelo munyu'ol	Oro
	2.			
	3.			
	4.			

<u>Mujulisi</u>: Aridho ni kisoma me otiti rijanyu'ol/jakur.Penji jie kanitye ochowi dwoko muniango go, aka go, kwong mito pere, oyeyo nyithindho/nyathi pere bedo ikisoma me.

Nyingi mujulisi:			
Chingi mujulisi:		Ndelo dwe:	

Nyingi ngata tito kisoma:			
Chingi:		Ndelo dwe:	
Sikulu:			

APPENDIX D: INITIAL SCREENING FORM (1)

**Primary school code:** |____|____|		**Primary school name:**
**Screening ID:** |____|____|____|____|		**Date of screening:** |___|___|/|___|___|/|___|___| *day month year*
**Student's last name**		**Student's first name**
**Student's initials***	**Student's class**	**Date of birth** |___|___|/|___|___|/|___|___| *day month year*
**Age:** |____|____| **years**		**Gender:** ☐ **Male** ☐ **Female**
**Parent/guardian's last name**		**Parent/guardian's first name**

**Initial of last name, followed by initial of first name*

ASSESS DURING SCREENING INTERVIEW WITH PARENT/GUARDIAN		
**Selection criteria**	**Include**	**Exclude**
1. Age > 8 to < 14 years (boys); > 8 to < 12 years (girls)	☐ Yes	☐ No
2. Student enrolled at participating school in classes 1 – 7	☐ Yes	☐ No
3. Known allergy or history of adverse reaction to study medications:<br>*If present, indicate drug / reaction:*<br>☐ Sulfadoxine-pyrimethamine:________________________________<br>☐ Amodiaquine:____<br>☐ Dihydroartemisinin-piperaquine:___________________________	☐ No	☐ Yes
4. Onset of menstruation (girls)	☐ N/A<br>☐ No	☐ Yes
If any boxes in the "Exclude" column are ticked, exclude from the study. If not, proceed to the next section.		

INFORMED CONSENT DISCUSSION		
**Selection criteria**	**Include**	**Exclude**
5. Willingness of parents or guardians to provide informed consent	☐ Yes	☐ No
If the box in the "Exclude" column is ticked, exclude from the study. If not, proceed to the next section.		

**All INITIAL Screening criteria met?**	**ASSIGN STUDY ID**
☐ Yes *If yes, assign Study ID*<br>☐ No *If no, exclude from the study*	|____|____|____|____|____|

**Completed by:**	**Signature:**

*First Entry*______________*Date*________________
*Second Entry*______________*Date*________________

UMSP / LSHTM / VCD
IPT in schoolchildren: Comparison of antimalarial regimens

INITIAL SCREENING FORM (2)

**Primary school code:** |____|____|	**Primary school name:**
**Study ID:** |_____|_____|_____|_____|_____|	**Date of screening:** |___|___|/|___|___|/|___|___| *day month year*
**Student's last name**	**Student's first name**
**Parent/guardian's last name**	**Parent/guardian's first name**

HOUSEHOLD INFORMATION

**Home parish:**	**LC1/village:**
**Home address and localizing features:**	
**Phone number**: ☐ Yes ☐ No<br>If yes: Phone number (s) and the owner (s):	

BED NET INFORMATION

**Does your household have any mosquito nets that can be used while sleeping?**	☐ Yes<br>☐ No *If no, skip to end.*
**How many mosquito nets does your household have?**	|____|____| nets
**When you got the net, was it already factory-treated with an insecticide to kill or repel mosquitoes?**	☐ Yes<br>☐ No<br>☐ Not sure
**Since you got the mosquito net, was it ever soaked or dipped in a liquid to repel mosquitoes or bugs?**	☐ Yes<br>☐ No<br>☐ Not sure
**How long ago was the net last soaked or dipped?**<br>*Record number of months if < 24 months.*<br>*If less than 1 month ago, record '00' months.*<br>*If less than 2 years ago, record number of months ago.*<br>*If '12 months ago' or '1 year ago,' probe for exact number of months.*<br>*If > 24 months ago, record number of years.*	|____|____| months ago<br>|____|____| years ago

**Completed by:**	**Signature:**

First Entry_____________Date_______________
Second Entry_____________Date_______________

APPENDIX E: CLINICAL SCREENING FORM

**Primary school code:** |____|_____|	**Primary school name:**
**Screening ID:** |_____|_____|_____|_____|	**Date of screening:** |____|____|/|____|____|/|____|____| *day month year*
**Study ID:** |_____|_____|_____|_____|_____|	**Student's initials***
**Student's last name**	**Student's first name**

ASSESS DURING SCREENING INTERVIEW		
**Selection criteria**	**Include**	**Exclude**
6. Onset of menstruation (girls)	☐ N/A<br>☐ No	☐ Yes
If the box in the "Exclude" column is ticked, exclude from the study. If not, proceed to the next section.		

ASSENT DISCUSSION		
**Selection criteria**	**Include**	**Exclude**
7. Willingness of student to provide assent	☐ Yes	☐ No
If the box in the "Exclude" column is ticked, exclude from the study. If not, proceed to the next section.		

CLINICAL SCREENING		
**Selection criteria**	**Include**	**Exclude**
8. Fever (≥ 37.5°C axillary) or history of fever in the previous 24 hours	☐ No	☐ Yes
9. Evidence of severe malaria or danger signs<br>*If " YES" indicate criteria. If "NO", leave blank.*<br>☐ Unarousable coma *(if after convulsion, > 30 min)*<br>☐ Repeated convulsions *(> 2 within 24 h)*<br>☐ Recent convulsions *(1-2 within 24 h)*<br>☐ Altered consciousness *(confusion, delirium,, coma)*<br>☐ Lethargy<br>☐ Unable to drink or breast feed<br>☐ Vomiting everything<br>☐ Unable to stand/sit due to weakness<br>☐ Respiratory distress *(labored breathing at rest)*<br>☐ Jaundice *(yellow coloring of eyes)*	☐ No	☐ Yes
10. Ongoing antimalarial treatment	☐ No	☐ Yes
11. Haemoglobin < 7.0 g/dL	☐ No	☐ Yes
12. Parasite density ≥ 10,000/ul	☐ No	☐ Yes
If the box in the "Exclude" column is ticked, exclude from the study. If not, proceed to the next section.		

ENROLLMENT	
**All criteria for study inclusion met?**<br>☐ Yes<br>☐ No *If no, exclude from the study*	**Date of enrollment** *(date study begins)*<br>|___|___|/|___|___|/|___|___| *day month year*

**Completed by:**	**Signature:**

First Entry______________Date________________
Second Entry______________Date________________

APPENDIX F: RESEARCH PARTICIPANT ASSENT FORM FOR CHILDREN

**Protocol Title:**	**IPT in school children: Comparison of the efficacy, safety, and tolerability of antimalarial regimens**
**Site of Research:**	Tororo, Uganda
**Principal Investigator**:	Sarah Staedke
**Date:**	17 March 2008

- I am being asked to decide if I want to be in this research study.
- I may be given medicine to treat malaria even if I am not feeling sick today.
- I know that I will have to see the study doctors at least 8 times over the next 6 weeks. The doctors will talk to me, ask me questions, and examine me.
- I know I will have a few drops of blood drawn at least 6 times over the next 6 weeks.
- I know that if I get sick during the next 6 weeks I should seek care and let my teachers and the study team know that I am sick. I may come to school to see the study doctors, or go to the health center or hospital.
- I asked and got answers to my questions. I know that I can ask questions about this study at any time.
- I know that I can stop being in this study at anytime without anyone being mad at me.

Mark one box with X:

**I DO CONSENT:**	☐	**I hereby agree to take part in this study**
**I DO <u>NOT</u> CONSENT:**	☐	**I do not wish to take part in this study**

Child's name		
Childs's Signature or Mark		Date:

Witness: I hereby confirm that the study has been explained to the child. All questions (if any) have also been answered to his/her satisfaction, and he/she has, of his own free will, consented to take part in the study.

Name of Witness:			
Signature of Witness:		Date:	

Name of person explaining study:			
Signature:		Date:	
School:			

APPENDIX F: FORMI MAYEROK PANYITHINDHO BEDO IMENYO TIENDI TWOO

**Nyinge Maradier:**	**IPT inyithidho masikulu: ki piima tich payBero, koodi tich payath gi chik mere**
**Kama lebedoiye:**	Tororo, Uganda
**Jadwar tiende madwong:**	Sarah Staedke
**Ka:**	17 March 2008

- Ikwayan yero kamito bedo ikisoma me.
- Inyalo miyan yath ma botho musuja makada ne'nde akawinji ni'atwoo konon.
- Angeyo ni ale neno lactar makisoma me kada di8 isabiti 6. Lactar la luwo kodan, penjan, kanyachiel gi kipiima an.
- Angeyo ni ale wodho remo manok moro di5 isabiti 6 mabino.
- Angeyo ni ka anwango twoo ihongo masabiti 6 me aripo limo kony gimiyo jufonji Paran koodi jumatimo kisoma me 'ngeyo ni atwoo. Anyalo bino isikulu lactar ma kisoma, kosa kidho I'od boyh kosa idwaliro.
- Apenjere aka alimo radwok ma penji paran. Angeyo ni anyalo penjo penji ma makere koodi kisoma me sawa morojie.
- Angeyo ni anyalo chungo /weyo bedo ikisoma me mungoye ngata oger ran.

Kethi ranyuthi isanduku achieli gi chali me X:

**AYERE:**	☐	**Ayere ka weyo nyathparan/nyithindho bedo ikisoma me**
**AKIYERE:**	☐	**Akiyere nyathparan/nyithidho bedo ikisoma me**

Nyingi nyath			
Chingi / nyathi		Ndelo dwe:	

Mujulisi: Aridho ni kisoma me otiti rijanyu'ol/jakur.Penji jie kanitye ochowi dwoko muniango go, aka go, kwong mito pere, oyeyo nyithindho/nyathi pere bedo ikisoma me.

Nyingi mujulisi:			
Chingi mujulisi:		Ndelo dwe:	

Nyingi ngata tito kisoma:			
Chingi:		Ndelo dwe:	
Sikulu:			

**APPENDIX G: CLINICAL RECORD FORM (1)**	**School class:** |_____|	**Primary school name:**
**Student Initials:**	**Study Number:** |_____|_____|_____|_____|_____|	**Date enrolled (Day 0):** |____|____|/|____|____|/|____|____| *day month year* **Weight** *(kg):*
**Age:** |_____|_____| years	**Gender:** ☐ Male ☐ Female	**Known drug allergies** ☐ No ☐ Unknown ☐ Yes *If yes, describe:*
"Does your household own a bed net?" ☐ Yes ☐ No ☐ Unknown		***"Did you sleep under a bed net last night?"*** ☐ Yes ☐ No ☐ Not applicable

SYMPTOM RECORD (Rank on scale of 0-4: absent = 0; mild = 1; moderate = 2; severe = 3, life-threatening = 4, N/A = unable to assess) **EXTRA FORM USED; YES —NO—**

	**DAY 0**	**DAY 1**	**DAY 2**	**DAY 3**	**DAY 7**	**DAY 14**	**DAY 28**	**DAY 42**	**DAY __**	**DAY __**	**DAY __**
**DATE**											
Outside treatment *(Y/N)**											
Fever in past 24h *(Y/N)*											
Weakness/fatigue Muscle/joint aches											
Headache											
Anorexia											
Nausea											
Vomiting											
Abdominal pain											
Diarrhea											
Cough											
"Flu"											
Pruritis											
Skin rash											
Tinnitus											
Other_______________											
Other_______________											
AE reported† *(Y/N)*											
Initials											

**** If yes, record treatment on CLINICAL RECORD FORM (4)***
† Notify Kampala core facility immediately of all serious adverse events.

**CLINICAL RECORD FORM (2)**	**Student Initials:**	**Study Number:** |_____|_____|_____|_____|_____|	**Date enrolled (Day 0):** |____|____|/|____|____|/|____|____| *day* *month* *year*

PHYSICAL EXAM RECORD (Rank on scale of 0-4: normal = 0; mild abnormality = 1; moderate = 2; severe = 3, life-threatening = 4, N/A = unable to assess)

	**DAY 0**	**DAY 1**	**DAY 2**	**DAY 3**	**DAY 7**	**DAY 14**	**DAY 28**	**DAY 42**	**DAY __**	**DAY __**	**DAY __**
**DATE**											
Temperature *(ºC)*											
Dehydration											
Jaundice											
Chest											
Abdomen											
Skin											
Tablet test											
Other__________											
Other__________											
AE reported† *(Y/N)*											
**ABNORMAL EXAM RECORD**											
If abnormality noted on physical exam, describe all physical findings for the abnormal exam											
**Initials**											

† Notify Kampala core facility immediately of all serious adverse events.

**CLINICAL RECORD FORM (3)**	**Student Initials:**	**Study Number:** |_____|_____|_____|_____|_____|	**Date enrolled (Day 0):** |____|____|/|____|____|/|____|____| *day* *month* *year*

LABORATORY RECORD

	DAY 0	DAY 1	DAY 2	DAY 3	DAY 7	DAY 14	DAY 28	DAY 42	DAY __	DAY __
**DATE**										
Parasite density *(asexual parasites/ul)*										
Species										
Gametocyte density *(gametocytes/ul)*										
Haemoglobin† *(g/dL)* [grade]	[ ]							[ ]		
Stool sample										
**Initials**										

† Any hemoglobin ≤ 5 g/dl measured after Day 0 is a serious AE. Notify Kampala core facility immediately of all serious adverse events.

RECORD OF ADDITIONAL MEDICATIONS GIVEN DURING STUDY

Medication (a)	Indication (b)	Dose (c)	Duration (d)	Date started (e)
**1.**				
**2.**				
**3.**				
**4.**				
**5.**				
**6.**				
**7.**				

CLINICAL RECORD FORM (4)	Student Initials:	Study Number: |_____|_____|_____|_____|_____|	Date enrolled (Day 0): |____|____|/|____|____|/|____|____| *day* *month* *year*

RECORD OF MEDICATIONS GIVEN OUTSIDE OF THE STUDY

Medication (a)	Indication (b)	Dose (c)	Duration (d)	Date started (e)
1.				
2.				
3.				
4.				

CLASSIFICATION OF OUTCOME

☐ **Clinical failure**	**Day of failure** |_____|_____|	*If clinical failure, indicate reason:*<br>☐ **Development of danger signs or severe malaria on Days 0 to 42 in the presence of parasitemia *If yes, specify criteria*:____________________<br>☐ **Temperature ≥ 37.5°C (A), or history of fever in previous 24 hours, on Days 3 to 42 in the presence of parasitemia
☐ **Parasitological failure**	**Day of failure** |_____|_____|	*If parasitological failure, indicate reason:*<br>☐ **Development of hyperparasitemia (≥ 10,000/ul) on Days 1 to 42<br>☐ Presence of parasitemia on Days 4 to 41 and axillary temperature < 37.5°C (A), without previously meeting any of the criteria of clinical failure<br>☐ **Presence of parasitemia on Day 42 and axillary temperature < 37.5°C (A), without previously meeting any of the criteria of clinical failure
☐ **ACPR**		Defined as: Absence of parasitemia on Day 42 irrespective of temperature without previously meeting any of the criteria for clinical failure or parasitological failure
☐ **Withdrawn**	**Day withdrawn** |_____|_____|	*If withdrawn, indicate reason:*<br>☐ Consent to participate withdrawn<br>☐ Lost to follow-up
☐ **No efficacy outcome**		*If no efficacy outcome, indicate reason:*<br>☐ Use of antimalarial drugs outside of the study protocol<br>☐ Incomplete treatment with study medications

** Requires rescue therapy

APPENDIX H. Guidelines for Grading Patient Symptoms, signs and laboratory findings

Table A. Guidelines for grading patient symptoms

	Grade 1<br>MILD	Grade 2<br>MODERATE	Grade 3<br>SEVERE	Grade 4<br>LIFE THREATENING
**Subjective fever in the past 24 h**	N/A	Present (Yes)	N/A	N/A
**Weakness**	Mild decrease in activity; For children – weak, but still playing	Moderate decrease in activity; For children – weak, and playing limited	Not participating in usual activities; For children – not playing	Prostration
**Muscle and/or joint aches***	Mild and/or localized complaints	Diffuse complaints	Objective weakness; function limited	N/A
**Headache***	Mild, no treatment required	Transient, moderate; treatment required	Severe, constant; requires narcotic therapy	Intractable; requires repeated narcotic therapy
**Anorexia**	Decreased appetite, but still taking solid food	Decreased appetite, avoiding solid food but taking liquids	Appetite very decreased; Refusing to breast feed, no solids or liquids taken (< 2 years ≤ 12 hr; > 2 years ≤ 24 hr)	Appetite very decreased; Refusing to breast feed, no solids or liquids taken (< 2 years > 12 hr; > 2 years > 24 hr)
**Nausea***	Mild, transient feeling of impending vomiting; maintains reasonable intake	Moderate and/or constant feeling of impending vomiting; intake decreased	Severe, constant feeling of impending emesis; intake decreased significantly	N/A
**Vomiting**	1 episode per day	2-3 episodes per day	Orthostatic hypotension or IV fluids required	Hypotensive shock or nrolment ation required for IV fluid therapy
**Abdominal pain***	Mild (1-3 on a scale of 1 to 10)	Moderate (4-6 on a scale of 1 to 10)	Moderate to severe (≥ 7 on a scale of 1 to 10)	Severe – nrolment at for treatment
**Diarrhea**	Transient 3-4 loose stools/day	5-7 loose stools/day	Orthostatic hypotension or > 7 loose stools/day or IV fluids required	Hypotensive shock or nrolment ation for IV fluid therapy required
**Cough**	Transient / intermittent	Persistent / constant	Uncontrolled	Cyanosis, stridor, severe shortness of breath
**Pruritis**	Transient pruritis	Pruritis that disturbs sleep	Severe, constant pruritis, sleep disturbed	N/A
**Tinnitus***	Mild, transient ringing or roaring sound	Moderate, persistent ringing or roaring sound	Severe ringing or roaring sound with associated hearing loss	N/A
**Behavioural changes**	Mild difficulty concentrating; mild confusion or agitation; activities of daily living unaffected; no treatment	Moderate confusion or agitation; some limitation of activities of daily living; minimal treatment	Severe confusion or agitation; Needs assistance for activities of daily living; therapy required	Toxic psychosis; nrolment ation required
**"Flu" (viral URI)**	Mild nasal congestion, mild rhinorrhea	Moderate nasal congestion, moderate rhinorrhea	N/A	N/A
**Allergic reaction**	N/A	N/A	Urticaria	Severe urticaria anaphylaxis, angioedema
**Convulsion**	N/A	N/A	Localized or generalized seizure	Status epilepticus
*** Assess only in children ≥ 3 years of age. Answer N/A for younger children and those unable to answer.**				

Reference – Based on WHO Toxicity Grading Scale for Determining the Severity of Adverse Events

Table B. Guidelines for Physical Examination

**Dehydration**	Assess skin touch and turgor, mucous membranes, eyes, crying, fontanelle, pulse, urine output
**Jaundice**	Assess for yellowing of the sclera. Also evaluate the palpepral conjunctiva, lips, and skin.
**Chest**	Observe the rate, rhythm, depth, and effort of breathing. Check the patient's colour for cyanosis.<br><br>The maximum acceptable respiratory rate by age: < 2 months = 60, 2-12 months = 50, 1-5 years = 40, above 5 years = 30.<br><br>Inspect the neck for the position of the trachea, for supraclavicular retractions, and for contraction of the sternomastoid or other accessory muscles during inspiration.<br><br>Auscultate the anterior and posterior chest for normal breath sounds and any adventitious sounds (crackles or rales, wheezes, and rhonchi). *Crackles are intermittent, non-musical, fine or coarse sounds that may be due to abnormalities of the lungs (pneumonia, fibrosis, early congestive heart failure) or airways (bronchitis or bronchiectasis). Wheezes are high-pitched and result from narrowed airways. Rhonchi are relatively low-pitched and suggest secretions in large airways.*<br><br>If abnormalities are identified, evaluate for transmitted voice sounds. In addition, palpate the chest to assess for tactile fremitus, and percuss the chest to assess for areas of dullness. *Normal, air-filled lungs emit predominantly vesicular breath sounds, transmit voice sounds poorly with "ee" = "ee", and have no tactile fremitus. Airless lung, as in lobar pneumonia, emits bronchial breath sounds, transmits spoken words clearly with "ee" = "aay" (egophany), and has an increase in tactile fremitus.*
**Abdomen**	Inspect and ausculate the abdomen. Listen for bowel sounds in the abdomen before palpating it. Palpate the abdomen in all 4 quadrants lightly and then deeply. Assess the size of the liver and spleen. To assess for peritoneal inflammation, look for localised and rebound tenderness, and voluntary or involuntary rigidity.
**Skin**	Inspect the skin for colour, turgor, moisture, and lesions. If lesions are present, note their location and distribution (diffuse or localised), arrangement (linear, clustered, annular, dermatomal), type (macules, papules, vesicles) and colour.
**Tablet test**	For children $\geq$ 9 months of age, ask the patient to pick a tablet (or equivalent object) up off a flat surface using the thumb and index finger of their dominant hand. *This tests for co-ordination of the upper extremity assessing the function of the motor system, cerebellar system, vestibular system (for coordinating eye and body movements) and the sensory system, for position sense. When testing small children, be aware that they will likely attempt to put the object into their mouth.*

Table C. Grading Physical Examination Findings

	Grade 1<br>MILD	Grade 2<br>MODERATE	Grade 3<br>SEVERE	Grade 4<br>LIFE-THREATENING
**Temperature * (axillary)**	37.5-37.9°C	38.0-39.5°C	> 39.5°C	Sustained fever, equal or greater than 40.0°C for longer than 5 days
**Dehydration**	Less than 2 of the following:<br>Restless, irritable<br>Sunken eyes<br>Drinks eagerly, thirsty<br>Skin pinch goes back slowly	2 of the following:<br>Restless, irritable<br>Sunken eyes<br>Drinks eagerly, thirsty<br>Skin pinch goes back slowly	Two of the following:<br>Lethargic or unconscious<br>Sunken eyes<br>Not able to drink or drinking poorly<br>Skin pinch goes back very poorly	Two of the following + shock:<br>Lethargic or unconscious<br>Sunken eyes<br>Not able to drink or drinking poorly<br>Skin pinch goes back very poorly
**Jaundice**	Slight yellowing of sclera and conjunctiva	Moderate yellowing of sclera and conjunctiva, yellowing of mucous membranes	Severe yellowing of sclera and conjunctiva, yellowing of skin	N/A
**Chest**	Mildly increased RR (for age, temperature), transient or localised adventitious sounds	Moderately increased RR, diffuse or persistent adventitious sounds	Rapid RR (< 2 months > 60, 2-12 months > 50, 1-5 years > 40, adults > 30)* nasal flaring, retractions	Cyanosis
**Abdomen**	Normal bowel sounds, mild localised tenderness, and/or liver palpable 2-4 cm below the right costal margin (RCM), and/or spleen palpable, and/or umbilical hernia present	Normal or mildly abnormal bowel sounds, moderate or diffuse tenderness; and/or mild to moderately enlarged liver (4-6 cm below the RCM) and/or spleen palpable up to half-way between umbilicus and symphysis pubis	Severely abnormal bowel sounds, severe tenderness to palpation. Evidence of peritoneal irritation and/or significant enlargement of liver (> 6 cm below the RCM) and/or spleen palpable beyond half-way between umbilicus and symphysis pubis	Absent bowel sounds. Involuntary rigidity
**Skin†**	Localised rash, erythema, or pruritis	Diffuse, maculopapular rash, dry desquamation	Vesiculation, moist desquamation, or ulceration	Exfoliative dermatitis, mucous membrane involvement or erythema multiforme or suspected Stevens-Johnson or necrosis requiring surgery

Table C. Grading Physical Examination Findings (continued)

	**Grade 1 MILD**	**Grade 2 MODERATE**	**Grade 3 SEVERE**	**Grade 4 LIFE-THREATENING**
**Hearing**	*< 4 years: N/A*<br>≥ 4 years: Decreased hearing in one ear	*< 4 years: N/A*<br>≥ 4 years: Decreased hearing in both ears or severe impairment in one ear	*< 4 years: Any evidence of hearing impairment*<br>≥ 4 years: Severe impairment in both ears	N/A
**Tablet test**	Difficulty grasping tablet but able to pick up	Unable to pick up tablet without dropping	Unable to grasp tablet	N/A
**Clinical symptoms / sign *(not otherwise specified)***	No treatment required; monitor condition	Treatment required	Requires treatment and possible hospitalisation	Requires active medical intervention, hospitalisation, or hospice care

) Reference – The Harriet Lane Handbook, 15th edition, 2000
† Reference – WHO Toxicity Grading Scale for Determining the Severity of Adverse Events

TABLE D. Guidelines for Grading of Laboratory Abnormalities

	**Grade 1 MILD**	**Grade 2 MODERATE**	**Grade 3 SEVERE**	**Grade 4 LIFE-THREATENING**
**Haemoglobin** *(g/dL)*	9.0 – 9.9	7.0 – 8.9	5.0 – 6.9	< 5.0

Reference – The Harriet Lane Handbook, 15th edition, 2000†
Reference – WHO Toxicity Grading Scale for Determining the Severity of Adverse Events

APPENDIX I. Weight-based administration of study medications

Table 1. Weight-based administration for SP group

Weight	SP	Placebo		
	Day 0	Day 0	Day 1	Day 2
8 – 10 kg	½	½	½	½
11 – 14 kg	1	½	½	½
15 – 20 kg	1	1	1	1
21 – 22 kg	1½	1	1	1
23 – 30 kg	1½	1½	1½	1½
31 – 35 kg	2	1½	1½	1½
36 – 37 kg	2	2	2	2
38 – 40 kg	2	2½	2½	2½
41 – 49 kg	3	2½	2½	2½
50 kg and over	3	3	3	3

Table 2. Weight-based administration for AQ+SP group

Weight	SP	AQ		
	Day 0	Day 0	Day 1	Day 2
8 – 10 kg	½	½	½	½
11 – 14 kg	1	½	½	½
15 – 20 kg	1	1	1	1
21 – 22 kg	1½	1	1	1
23 – 30 kg	1½	1½	1½	1½
31 – 35 kg	2	1½	1½	1½
36 – 37 kg	2	2	2	2
38 – 40 kg	2	2½	2½	2½
41 – 49 kg	3	2½	2½	2½
50 kg and over	3	3	3	3

Table 3. Weight-based administration of study medications for DP group

Weight (kg)	Dihydroartemisinin-piperaquine (DP 40mg/320mg)				
	Day 0	Day 1	Day 2	Total DHA dose (mg/kg)	Total PQ dose (mg/kg)
5	¼	¼	¼	6.0	48.0
6	½	½	½	10	80.0
7	½	½	½	8.6	68.6
8	½	½	½	7.5	60.0
9	½	½	½	6.7	53.5
10	½	½	½	6.0	48.0
11	¾	¾	¾	8.2	65.5
12	¾	¾	¾	7.5	60.0
13	¾	¾	¾	6.9	55.4
14	¾	¾	¾	6.4	51.4
15	1	1	1	8.0	64.0
16	1	1	1	7.5	60.0
17	1	1	1	7.1	56.5
18	1	1	1	6.7	53.3
19	1	1	1	6.3	50.5
20	1 ¼	1 ¼	1 ¼	7.5	60.0
21	1 ¼	1 ¼	1 ¼	7.1	57.1
22	1 ¼	1 ¼	1 ¼	6.8	54.5
23	1 ¼	1 ¼	1 ¼	6.5	52.3
24	1 ½	1 ½	1 ½	7.5	60.0
25	1 ½	1 ½	1 ½	7.2	57.6
26	1 ½	1 ½	1 ½	6.9	55.4
27	1 ½	1 ½	1 ½	6.7	53.3
28	1 ½	1 ½	1 ½	6.4	51.4
29	1 ¾	1 ¾	1 ¾	7.2	57.9
30	1 ¾	1 ¾	1 ¾	7.0	56.0
31	1 ¾	1 ¾	1 ¾	6.8	54.2
32	1 ¾	1 ¾	1 ¾	6.6	52.3
33	1 ¾	1 ¾	1 ¾	6.4	50.9
34	2	2	2	7.1	56.5
35	2	2	2	6.9	54.9
36	2	2	2	6.7	53.3
37	2	2	2	6.5	51.2
38	2	2	2	6.3	50.5
39	2	2	2	6.2	49.2
40	2	2	2	6.0	48.0

Table 4. Weight-based administration for Placebo group

Weight	Placebo		
	**Day 0**	**Day 1**	**Day 2**
8 – 10 kg	½	½	½
11 – 14 kg	½	½	½
15 – 20 kg	1	1	1
21 – 22 kg	1	1	1
23 – 30 kg	1½	1½	1½
31 – 35 kg	1½	1½	1½
36 – 37 kg	2	2	2
38 – 40 kg	2½	2½	2½
41 – 49 kg	2½	2½	2½
50 kg and over	3	3	3

APPENDIX J: ACCEPTABILITY QUESTIONNAIRE

**Study Number:** |_____|_____|_____|_____|_____|	**Date:** |____|____| / |____|____| / |____|____| day month year

**I would like to ask you some questions about the 3-day treatment you received this week.**	
1. How many tablets did you take at one time on the last day of treatment?	|____| full tablets |____| fractions<br>☐ Don't know
2. What did you think about the taste of the tablets?	☐ Poor<br>☐ Fair<br>☐ Good<br>☐ Excellent<br>☐ Don't know
3. How would you describe the taste of the tablets?<br>Please describe	
4. What did you think about the color of the tablets?	☐ Poor<br>☐ Fair<br>☐ Good<br>☐ Excellent<br>☐ Don't know
5. Did you have any problems during the 3 days that you received the treatment?	☐ Yes<br>☐ No<br>☐ Don't know
6. If so, please describe the problems:	
7. Did you have any problems during the 4 days after you received the treatment?	☐ Yes<br>☐ No<br>☐ Don't know
8. If so, please describe the problems:	
9. Would you be willing to take the tablets during every school term (three times a year)?	☐ Yes, very willing<br>☐ Yes, willing<br>☐ Don't know<br>☐ No, not willing<br>☐ No, absolutely not
10. Additional comments:	

Completed by:____

**APPENDIX K. ADVERSE EVENT REPORT FORM**	**Student Initials:**	**Study Number:** |_____|_____|_____|_____|_____|	**Date enrolled (Day 0):** |____|____|/|____|____|/|____|____| *day* *month* *year*

	Complete on day first reported			***Complete on day first reported and update as needed***			***Complete on final day***		
**Event description (a)**	**Date of event onset (b)**	**Date event reported (c)**	**Initials of person reporting**	**Maximum severity* (d)**	**Maximum relationship† (e)**	**Serious? ‡ *(Y/N)* (f)**	**Episodic? *(Y/N)* (g)**	**Outcome †† (h)**	**Date event resolved‡‡ (i)**
**1.**									
**2.**									
**3.**									
**4.**									
**5.**									
**6.**									
**7.**									
**8.**									
**9.**									
**10.**									
**11.**									
**12.**									
**13.**									
**14.**									
**15.**									

*** Severity:** Rank on scale of 1-4: mild = 1; moderate = 2; severe = 3, life-threatening = 4
† **Relationship:** Rank on scale of 0-4: none = 0; unlikely = 1; possible = 2; probable = 3; definite = 4
‡ **Serious:** Criteria for SAE: fatal, life-threatening, results in or prolongs hospitalization, results in significant or persistent disability or capacity requires medical / surgical intervention to prevent serious outcome. <u>If serious, report to Kampala core facility staff immediately.</u>
†† **Outcome:** Rank on scale of 1-5: resolved without sequelae = 1; resolved with sequelae = 2; AE still present at study end/discontinuation, but improving = 3; subject died = 4; unknown = 5
‡‡ **Date event resolved:** Complete on Day 42 – If AE still ongoing at end of follow-up, indicate in question (h).

**APPENDIX K. SERIOUS ADVERSE EVENT FORM – INITIAL REPORT (1)**	**Date of SAE Report:** |___|___|/|___|___|/|___|___| *day month year*
**Study Number:** |___|___|___|___|___|	**Date enrolled:** |___|___|/|___|___|/|___|___| *day month year*
**Age:** |___|___| years	**Gender:** ☐ Male ☐ Female

Event description:____
(symptom, sign, or laboratory abnormality)

**Indicate reason for serious AE** *(tick all that apply):*<br>☐ Death<br>☐ Life-threatening<br>☐ Resulted in or prolonged hospitalization<br>☐ Required medical / surgical intervention to prevent serious outcome<br>☐ Resulted in significant / persistent disability or incapacity<br>☐ Other:____________________	**Date of event onset:**<br>|___|___|/|___|___|/|___|___| *day month year*<br>**Date of site awareness:**<br>|___|___|/|___|___|/|___|___| *day month year*	**Maximum event severity:**<br>☐ Mild<br>☐ Moderate<br>☐ Severe<br>☐ Life-threatening

EVENT SUMMARY *(include details of event, associated signs and symptoms, possible alternative etiologies, relevant past medical history, and medical management):*
____
____
____
____
____
____
____
____
____
____

**Study product name: BLINDED**	**Route of study product: ORAL**
**Dosing schedule at SAE onset:**	
**Date study product first started** *(Day 0)***:**<br>|__|__|/|__|__|/|__|__| *day month year*<br>**Relationship to study product:**<br>☐ None<br>☐ Unlikely<br>☐ Possible<br>☐ Probable<br>☐ Definite	**Date study product last taken prior to onset of SAE:**<br>|__|__|/|__|__|/|__|__| *day month year*<br>**If not associated, is event related to:**<br>☐ Study procedure<br>☐ Other condition or illness<br>☐ Other medication<br>☐ Other____________________________

SERIOUS ADVERSE EVENT FORM – INITIAL REPORT (2)

Date of SAE Report: |___|___|/|___|___|/|___|___|
day month year

Study Number: |____|____|____|____|____|

Study product status: *(tick all that apply)*

☐ No change in dose
☐ Study treatment held
☐ Study treatment discontinued permanently
☐ Other______________________________

Patient management: *(tick all that apply)*

☐ Patient hospitalized
☐ Blood transfusion given
☐ Intravenous fluids given
☐ Parenteral quinine given
☐ Other______________________________
☐ Other______________________________
☐ Other______________________________

RELEVANT LABORATORY TESTS

Test	Collection date *(dd/mm/yy)*	Result	Site normal range	Most recent value prior to SAE	Collection date *(dd/mm/yy)*
	|__|__|/|__|__|/|__|__|				|__|__|/|__|__|/|__|__|
	|__|__|/|__|__|/|__|__|				|__|__|/|__|__|/|__|__|
	|__|__|/|__|__|/|__|__|				|__|__|/|__|__|/|__|__|
	|__|__|/|__|__|/|__|__|				|__|__|/|__|__|/|__|__|

RELEVANT DIAGNOSTIC TESTS

Test	Collection date *(dd/mm/yy)*	Results/Comments
	|__|__|/|__|__|/|__|__|	
	|__|__|/|__|__|/|__|__|	
	|__|__|/|__|__|/|__|__|	

CONCOMITANT MEDICATIONS *(List relevant concomitant medications the subject was taking up to 1 month prior to SAE onset.)*

Medication	Start date *(dd/mm/yy)*	Stop date *(dd/mm/yy)*	Total daily dose	Indication	Suspect for SAE
	|__|__|/|__|__|/|__|__|	|__|__|/|__|__|/|__|__|			☐ Yes ☐ No
	|__|__|/|__|__|/|__|__|	|__|__|/|__|__|/|__|__|			☐ Yes ☐ No
	|__|__|/|__|__|/|__|__|	|__|__|/|__|__|/|__|__|			☐ Yes ☐ No
	|__|__|/|__|__|/|__|__|	|__|__|/|__|__|/|__|__|			☐ Yes ☐ No
	|__|__|/|__|__|/|__|__|	|__|__|/|__|__|/|__|__|			☐ Yes ☐ No
	|__|__|/|__|__|/|__|__|	|__|__|/|__|__|/|__|__|			☐ Yes ☐ No

Outcome of event:

☐ Ongoing: [SAE Follow Up Report to be completed and sent at later date]
☐ Resolved without sequelae
☐ Resolved with sequelae ________________________
☐ Death

If resolved or died, indicate date: |___|___|/|___|___|/|___|___|
day month year

Completed by:
Name printed: ______________________ **Signature:** ______________________ **Date:** |___|___|/|___|___|/|___|___|
day month year

Investigator's
Name printed: ______________________ **Signature:** ______________________ **Date:** |___|___|/|___|___|/|___|___|
day month year
